# Pilot imaging study of *o*-BMVC foci for discrimination of indeterminate cytology in diagnosing fine-needle aspiration of thyroid nodules

**DOI:** 10.1038/s41598-021-02887-8

**Published:** 2021-12-06

**Authors:** Ting-Yuan Tseng, Shyang-Rong Shih, Cheng-Ping Wang, Shang-Jyun Lin, I.-Shiow Jan, Chiung-Lin Wang, Shin-Ya Liu, Cheng-Chung Chang, Pei-Jen Lou, Ta-Chau Chang

**Affiliations:** 1 Institute of Atomic and Molecular Sciences, Academia Sinica, Taipei, 106 Taiwan; 2 Division of Metabolism and Endocrinology, National Taiwan University Hospital, National Taiwan University College of Medicine, Taipei, 100 Taiwan; 3 Department of Otolaryngology, National Taiwan University Hospital, National Taiwan University College of Medicine, Taipei, 100 Taiwan; 4 Department of Laboratory Medicine, National Taiwan University Hospital, National Taiwan University College of Medicine, Taipei, 100 Taiwan; 5 Graduate Institute of Biomedical Engineering, National Chung-Hsing University, Taichung, 402 Taiwan

**Keywords:** DNA, Biomarkers, Cancer screening, Endocrine cancer

## Abstract

Fluorescence lifetime imaging microscopy of a fluorescence probe, 3,6-bis(1-methyl-2-vinylpyridinium) carbazole diiodide (*o*-BMVC), provides an objective method for preoperative diagnosis of fine-needle aspiration (FNA) of thyroid nodules. The key of this *o*-BMVC test of FNA smears is the measurement of the digital number of *o*-BMVC foci in the nucleus. Thus, there are three categories classified in the *o*-BMVC test, which are nondiagnostic for unsatisfactory samples, benign for less numbers of *o*-BMVC foci, and malignant for more numbers of *o*-BMVC foci. The discrimination of indeterminate (including atypia, follicular neoplasm, suspicious) cytology into benign or malignant cases can reduce diagnostic uncertainty and benefit clinical decision making. This pilot study strongly suggests that the *o*-BMVC test is an invaluable method for diagnosing FNA samples. Particularly, the combination of FNA cytology and the *o*-BMVC test holds great promise to improve the efficacy of diagnosis and reduce the healthcare costs.

## Introduction

Thyroid nodules are common in the adult population. The prevalence of thyroid nodules increases with age, from nearly 42% for younger population (< 40 years) to about 76% for older population (> 61 years)^1,2^. To evaluate thyroid cancer, ultrasound-guided fine-needle aspiration (FNA) cytology has been widely used for the diagnosis of thyroid nodules^3–5^. Because of the undetermined cases due to unsatisfactory or indeterminate samples, the Bethesda system provided six diagnostic categories for reporting thyroid cytopathology, including nondiagnostic, benign, atypia, follicular neoplasm, suspicious, and malignant categories, for the guidance of clinical decisions^6,7^. Approximately 15–30% of FNA samples are nondiagnostic (unsatisfactory)^4^ and nearly 10–25% of the remaining samples are indeterminate (including atypia, follicular neoplasm, and suspicious) cytology^8,9^. Generally, patients with a nondiagnostic result require repeated aspiration, while patients with an indeterminate result will probably be referred for surgical treatment^10^. Notably, the majority of surgical removals are proven to be unnecessary^8–11^.

Over the last decade, molecular testing based on gene expression was developed and could accurately classify more patients with indeterminate thyroid nodules as benign, which decreases the number of diagnostic surgeries and benefits the patient in terms of quality of life^8–12^. However, molecular testing is not easily available commercially, and the high costs (between $3000 and $5000 per test) limit its worldwide use^13^. Thus, developing a method for discriminating indeterminate cytology into benign or malignant cases and reducing the number of nondiagnostic cytology at a low cost is welcome and an urgent need.

Cancer is a universal disease and remains a major cause of death. Although the causes of cancers can be very diverse, the loss of genomic integrity is a common hallmark of cancer^14,15^. Recently, the study of G-quadruplex (G4) structures in cells has received increasing attention^16–18^. G4 structures are four-stranded secondary structures formed by the stacking of G-quartets via a cyclic Hoogsteen hydrogen-bonding arrangement of four guanines under physiological conditions^19,20^. In addition to accumulating studies of the biological functions of G4 supporting the existence of G4 structures in vivo^21–23^, a number of bioimaging studies have provided convincing evidence to support the presence of G4 structures in cells^24–27^. Moreover, many more G4 foci are detected in cancer cells than in normal cells^28–30^, implying that the G4 DNA may act as a common marker of cancer cells.

Previously, a fluorescent probe, 3,6-bis(1-methyl-2-vinylpyridinium) carbazole diiodide (*o*-BMVC), was synthesized to stain G4 structures in cells^25,29^. There are two distinct advantages of this molecule: *o*-BMVC has a much better binding affinity to G4 DNA than duplex DNA by approximately two orders of magnitude, and it has longer fluorescent decay times upon interaction with G4 structures (≥ 2.4 ns) than other structures, such as single strands and duplexes (~ 1.2 ns). Fluorescence lifetime imaging microscopy (FLIM) was applied to visualize the G4 structures by separating the image into two channels: one color with a decay time of ≥ 2.4 ns and another color with a decay time of < 2.4 ns^25^. Of importance is that such binary images show more *o*-BMVC foci detected in six types of cancer cell lines than in three types of normal cell lines^29^. Further application of the *o*-BMVC test in the clinical examination of head and neck cancer showed that the average number of *o*-BMVC foci was 28.3 in 50 head and neck patients and 2.2 in 20 healthy volunteers, suggesting that the *o*-BMVC test is useful for cancer diagnosis. Here, the key of the *o*-BMVC test is the digital number of *o*-BMVC foci measured in the nucleus of FNA samples, which can simplify the diagnostic results of FNA cytology. Thus, it is important to evaluate whether the *o*-BMVC test of FNA biopsies can improve the efficacy of diagnosis of thyroid nodules by discriminating indeterminate cytology into benign or malignant cases and decreasing the number of nondiagnostic cytology.

## Results

### A pilot study of the *o*-BMVC test for FNA of thyroid nodules

In this work, a pilot study of 318 patients with 327 FNAs collected from National Taiwan University Hospital (NTUH) was conducted to evaluate the potential of the *o*-BMVC test in the preoperative diagnosis of thyroid nodules between October 2018 to November 2019 (Supplementary Table S1). In addition, we collected 9 FNA samples for the follow-up examination from May 2020 to December 2020 (Supplementary Table S2). To compare the *o*-BMVC test with the standard method of cytological examination, the major portions of aspirates were first used for cytological examination, and the residual aspirates were then used for the *o*-BMVC test. The diagnostic results were obtained independently from cytological examination and the *o*-BMVC test for FNA smears of thyroid nodules.

**Figure 1:**
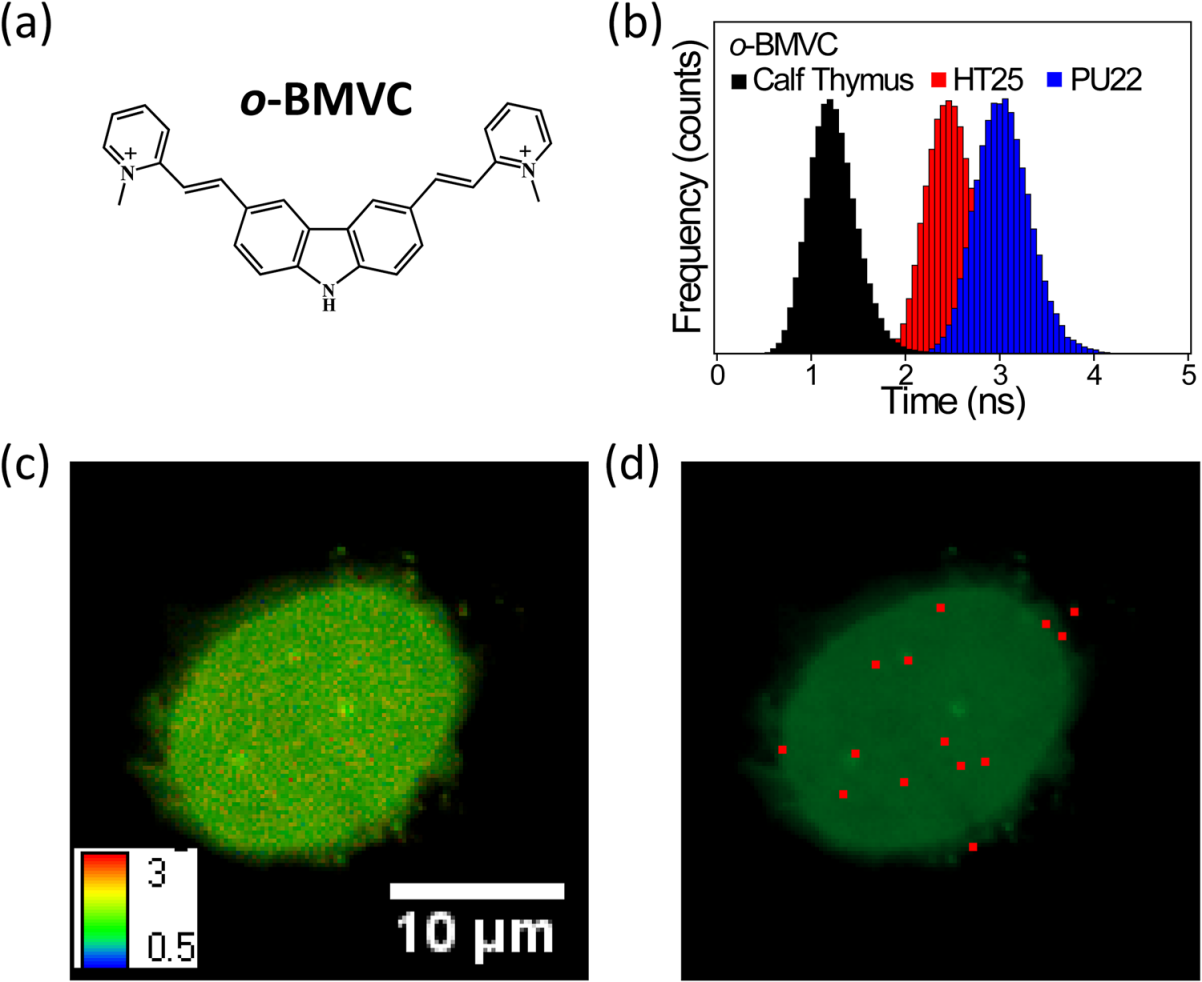
Binary FLIM image of *o*-BMVC foci. **(a)** Chemical structure of *o*-BMVC. **(b)** The histogram of the fluorescent decay time of *o*-BMVC upon interaction with calf thymus, HT25 (TAG_3_T_2_AG_3_T_2_AG_3_T_2_AG_3_TT), and PU22 (TGAG_3_TG_4_AG_3_TG_4_AA). The decay time of 2.4 ns was applied to distinguish duplex and G4 structures. **(c)** A typical FLIM image of a FNA smear of thyroid nodules stained by *o*-BMVC. **(d**) Using the Otsu method, the analyzed binary image was separated into two colors: red (decay time ≥ 2.4 ns) and green (decay time < 2.4 ns).

Here we briefly describe the *o*-BMVC foci in the nucleus. Figure 1a shows the chemical structure of *o*-BMVC, and Fig. 1b shows a histogram of the fluorescent decay time of *o*-BMVC upon interaction with duplexes of calf thymus and G4 structures of HT25 G4 (TAG_3_T_2_AG_3_T_2_AG_3_T_2_AG_3_TT) in the human telomere^31^ and PU22 (TGAG_3_TG_4_AG_3_TG_4_AA) in the human c-MYC promoter^32^. Such longer fluorescent decay time of *o*-BMVC upon interaction with G4 structures than non-G4 structures detected in 20 different G-rich sequences of G4 structures and 10 different sequences of non-G4 structures suggested that the decay time of 2.4 ns can be applied to distinguish G4 and non-G4 structures^29^. In the study of FNA samples, 10 FLIM images of *o*-BMVC staining per FNA smear were generally collected. Figure 1c shows a typical FLIM image of a FNA smear stained by *o*-BMVC. Using the Otsu threshold method^29,33^, the FLIM image was analyzed and separated into two channels: red (decay time ≥ 2.4 ns) and green (decay time < 2.4 ns), as shown in Fig. 1d. The red spots were named *o*-BMVC foci and the number of *o*-BMVC foci in the nucleus was measured for cancer diagnosis.

**Figure 2:**
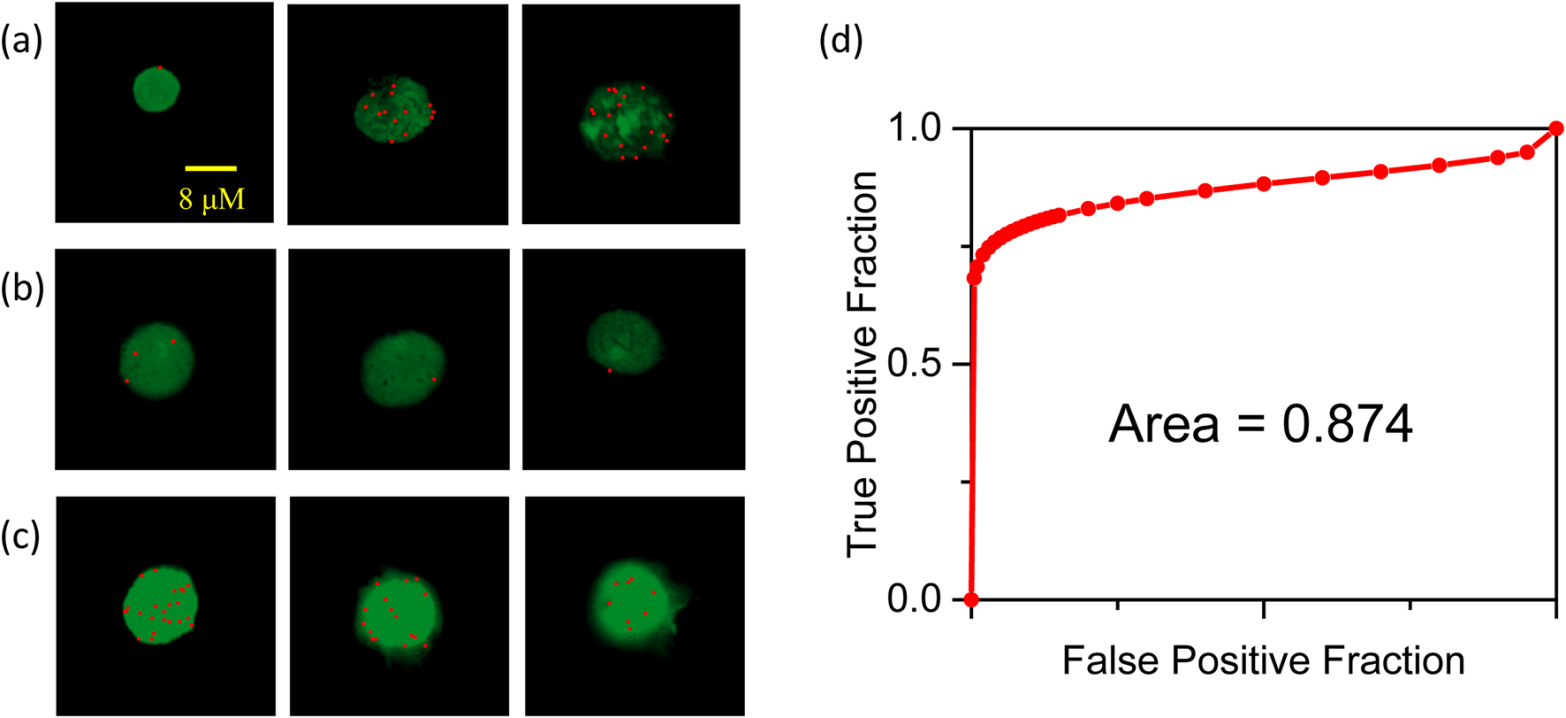
Binary FLIM images of the *o*-BMVC test for **(a)** nondiagnostic, **(b)** benign, and **(c)** malignant samples. **(d)** The receiver operating characteristic (ROC) curve obtained from the total number of the top three foci results compared to the results from pathological reports and the repeated cases of cytological reports.

We first define satisfactory images for the *o*-BMVC test. In this work, some spots with small size can be easily collected in the images. This is because FNA samples can be highly heterogeneous, including red blood cells, white blood cells, some overlapping cellular contents, and other contaminants. Such spots could mislead diagnostic results. To avoid such problem to the *o*-BMVC test, each image was first examined by the size of the nucleus (≥ 8 μm). In addition, we consider that broken nuclei are possibly associated with the opening of chromatin, which could have more *o*-BMVC foci. Figure 2a shows three images including one small and two broken nuclei. Such images were unsatisfactory (nondiagnostic) for the *o*-BMVC test.

After analyzing and examining the 10 images (each image displayed one cell nucleus), we found that more benign cells than cancer cells were collected from FNA of thyroid nodules for most cases. Here we used 3 qualified images with the highest numbers of *o*-BMVC foci in the nuclei for the *o*-BMVC test to differentiate malignant and benign nodules. Figure 2b shows three binary FLIM images of the *o*-BMVC test on an FNA smear collected from a 51-year-old female (#95). This case is classified as a benign thyroid because the numbers of *o*-BMVC foci in the nuclei are 3, 1, and 0. Figure 2c shows three images of the *o*-BMVC test on an FNA smear collected from a 75-year-old female (#72). Since the numbers of *o*-BMVC foci are 22, 11, and 8 measured in the nuclei, the total number of *o*-BMVC foci is 41. This case is classified as malignant because a threshold value of 20 based on receiver operating characteristic (ROC) curve analysis of the total number of the top three foci was obtained to differentiate malignant and benign nodules (Fig. 2d)^34^. The area under the ROC curve was 0.874, suggesting that the *o*-BMVC test provides reasonable accuracy for the detection of thyroid malignancy. Generally, it is easy to differentiate malignant cases from benign cases based on the total number of *o*-BMVC foci. However, several cases lacked satisfactory samples and some cases had a total number of *o*-BMVC foci > 20; particularly, one had a large number of *o*-BMVC foci and others had fewer than 5 foci. Such cases can be verified by examining the second FNA smear because each residual sample had at least two FNA smears in sample preparation for the *o*-BMVC test.

We further proposed an algorithm as an additional criterion for the *o*-BMVC test to differentiate malignant and benign nodules. A score is given based on the number of *o*-BMVC foci (n) in each nucleus: 0 for n < 6; 1 for n = 6 and 7; 2 for n = 8 and 9; and 3 for n > 9. Accordingly, we established simplified criteria for the diagnosis of thyroid nodules based on the total score (S) of the top three scores for each FNA sample, with S ≤ 3 for benign nodules and S > 3 for malignant nodules. An additional examination of the second FNA smear was conducted for the initial test with S = 3. Among the 327 FNA smears, the *o*-BMVC test showed 61 nondiagnostic, 236 benign, and 30 malignant cases. Supplementary Table S1 lists the results of FNA cytology, the *o*-BMVC test, and the pathology of the 327 thyroid nodules. In addition, Supplementary Fig. S1 shows the three images of 30 positive cases reported by the *o*-BMVC test.

In general, nondiagnostic cases should undergo repeated FNA examination, and benign cases may have a follow-up FNA examination. Here, we collected 9 repeated FNAs from May 2020 to December 2020 in NTUH. Supplementary Table S2 lists their results of FNA cytology and the *o*-BMVC test. Although the *o*-BMVC test classified 3 nondiagnostic cases in the initial report, these 9 repeated cases were all benign. Given that 34 out of 61 nondiagnostic cases were due to the contribution from small size of FNA nodules predominately in the images, where a typical case (#270) is shown in Supplementary Fig. S2, careful study by minimizing the contribution from small size of FNA nodules in the images may be useful to reduce the number of nondiagnostic cytology.

Furthermore, we assessed the 33 unused FNA smears (17 benign and 16 malignant samples) to examine the reproducibility of the *o*-BMVC test for thyroid nodules. Notably, these samples were prepared several months ago. In comparison with the previous results of the *o*-BMVC test, the consistency rate for two independent *o*-BMVC tests was 90.9% (30/33). Two cases (#65 and #71) were detected from positive to negative, while one case (#75) was detected from negative to positive. Given that the negative result of the *o*-BMVC test is likely due to the lack of FNA samples, these three cases were reported as positive in this work. The other 30 cases showed the same results as previous reports, suggesting that the *o*-BMVC test for FNA smears of thyroid nodules can be used as a send-out test for the diagnosis of FNA nodules. Again, no nondiagnostic case was reported in this late study.

### Comparison of the *o*-BMVC test for thyroid nodules to cytological examination

**Figure 3:**
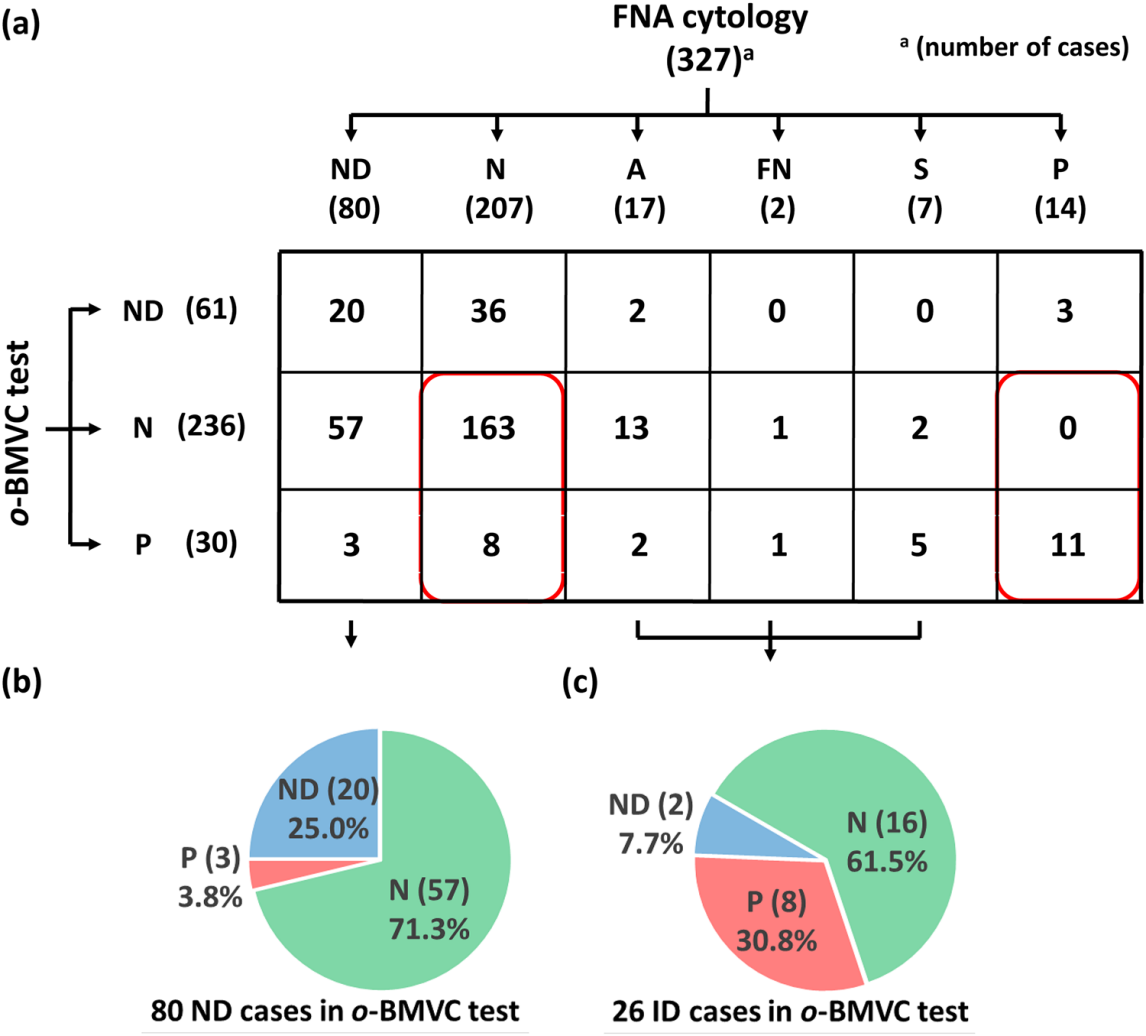
**(a)** The *o*-BMVC test results of thyroid nodules correlated with the cytological results. The *o*-BMVC test showed three categories: nondiagnostic (ND) for unsatisfactory sample, negative (N) for benign, and positive (P) for malignant, and no cytologically indeterminate (ID) cases, including atypia (A), follicular neoplasm (FN), and suspicious (S) categories for the diagnosis of thyroid nodules. The two red circles are determined cases reported by both methods. **(b)** The *o*-BMVC test could discriminate 75.0% of cytologically nondiagnostic cases into benign or malignant cases. **(c)** The *o*-BMVC test can discriminate 92.3% of cytologically indeterminate cases to benign or malignant cases.

Cytological examination of FNA is a well-established method for the preoperative diagnosis of thyroid nodules and can be used as the gold standard for diagnostic comparison. Figure 3 summarizes the *o*-BMVC test results correlated with the cytological results. Although the residual aspirates were used for the *o*-BMVC test, there were 61 nondiagnostic cases in the *o*-BMVC test. However, there were 80 nondiagnostic cases in the cytological examination. Of importance is that the *o*-BMVC test was able to discriminate 266 satisfactory cases into 236 benign cases and 30 malignant cases. Considering the 182 determined cases from both methods, the comparison of the *o*-BMVC test to cytological examination showed a very high consistency rate of 95.6% (174/182). These findings suggest that the *o*-BMVC test can be applied to diagnose thyroid nodules. Notably, there were 8 contradictory cases, which were negative from cytological examination but positive from the *o*-BMVC test. Such cases deserve immediately repeated examination.

In addition, the *o*-BMVC test discriminated 75.0% (60/80) of cytologically nondiagnostic cases and 92.3% (24/26) of cytologically indeterminate (including atypia, follicular neoplasm, and suspicious) cases into benign or malignant cases. The discrimination of the 60 cytologically nondiagnostic cases into 57 benign and 3 malignant cases suggests that the interval for repeated FNAs for these 57 benign cases can be extended, which can reduce unnecessary panic and costs. For the 3 malignant cases, they deserve immediately repeated examination. The discrimination of the 24 cytologically indeterminate cases into 16 benign and 8 malignant cases provides unambiguous results for clinical decision making.

### Pathological results of thyroid nodules

**Figure 4:**
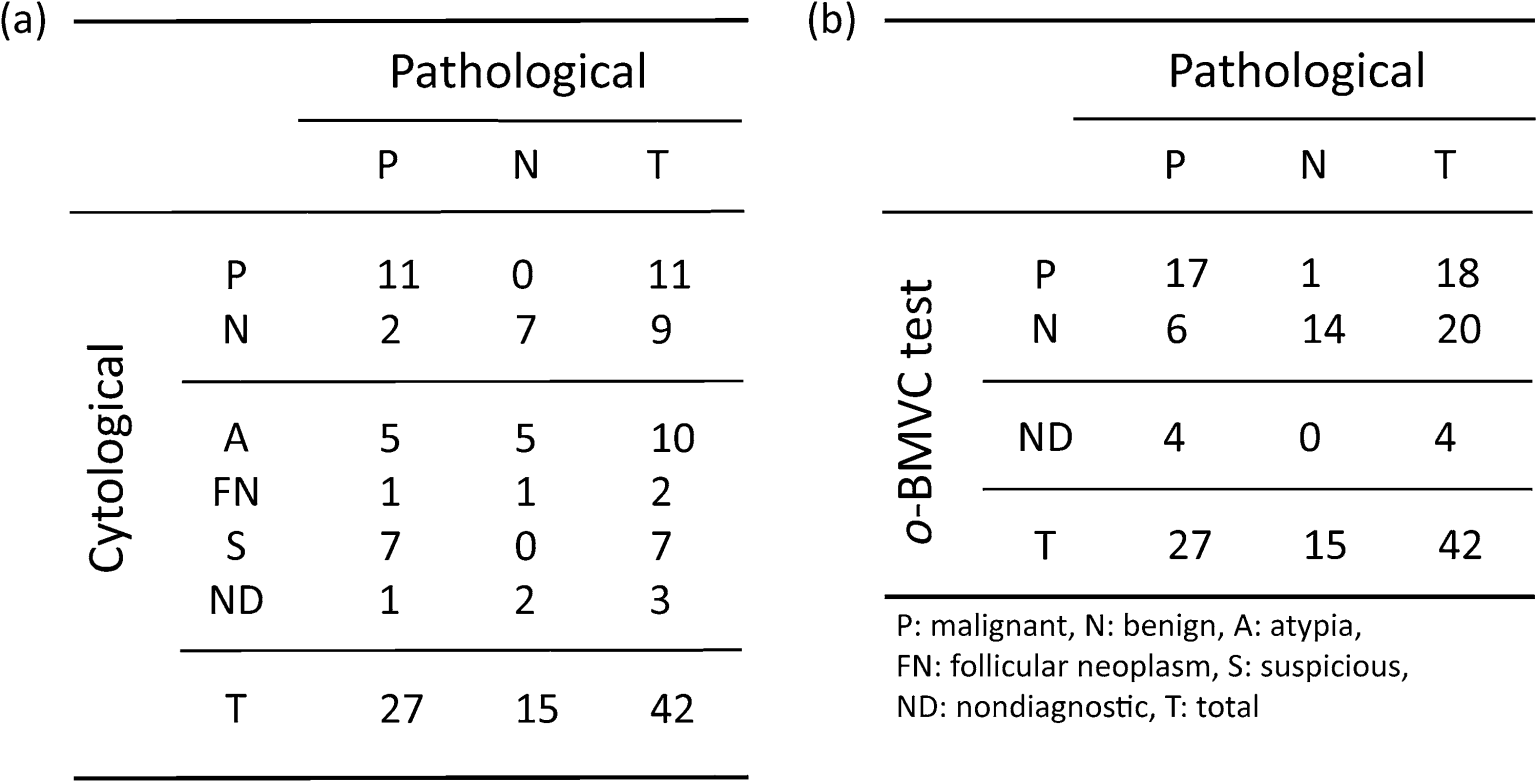
The cytological **(a)** and *o*-BMVC test **(b)** results of thyroid nodules correlated with the pathological results.

The definitive diagnosis of a thyroid patient was determined by the pathological report. Among the 327 thyroid nodules, there were 27 positive and 15 negative cases from pathological reports. Figure 4a shows the distribution of the pathological results in the cytological classifications. Because there were 3 nondiagnostic cases and 19 indeterminate cases from cytological reports, the limited data gave a diagnostic accuracy of 90.0% (18/20) for cytological examination. Figure 4b summarizes the correlation of the *o*-BMVC test results with the pathological results. In this study, there were 4 nondiagnostic cases from the *o*-BMVC test. Although the pathological results are limited, this pilot study shows a preliminary diagnostic accuracy of 81.6% (31/38), a sensitivity of 73.9% (17/23), a specificity of 93.3% (14/15), a positive predictive value (PPV) of 94.4% (17/18), and a negative predictive value (NPV) of 70% (14/20) for the *o*-BMVC test. Considering the two false-negative cases (#16 and # 124) from cytology, the *o*-BMVC test showed that the former one was positive and the latter one was also false-negative (Supplementary Table S1).

Among the 22 pathological results classified as cytologically undetermined nodules, the *o*-BMVC test showed 12 negative, 9 positive, and 1 nondiagnostic cases. There were 5 false-negative cases (#9, #80, #98, #137, and #147) and 1 false-positive case (#48) from the *o*-BMVC test (Supplementary Table S1). Without considering the 1 nondiagnostic case, the diagnostic accuracy of the *o*-BMVC test for the discrimination of cytologically undetermined cases is 71.4% (15/21), suggesting that the *o*-BMVC test is an exciting method developed for the diagnosis of thyroid nodules.

## Discussion

The use of an *o*-BMVC fluorescent probe to stain G4 structures initiates the *o*-BMVC test. The detection of more *o*-BMVC foci in cancer cells than in normal cells is critical for its use in cancer diagnosis^25,29^. The finding of more G4 foci in human cancers of the liver and stomach than in nonneoplastic tissue was previously reported using a G4-specific antibody, BG4, and immunofluorescence microscopy^28^. In addition, more G4 foci induced by pyridostatin molecules^35^ and UV irradiation^36^ are attributed to DNA damage, which can possibly lead to genomic instability and open chromatin and facilitate G4 formation from unprotected G-rich sequences. In this work, more *o*-BMVC foci were detected in some broken nuclei (Fig. 2a). Although broken nuclei were excluded in preoperative diagnosis, such images indeed support that the loss of chromatin integrity plays a role in the detection of more G4 foci.

Previously, we reported that the average numbers of *o*-BMVC foci in tissue biopsies from 50 head and neck cancer samples and in oral epithelial cells from 20 volunteers were 28.3 and 2.2, respectively^29^. Here, the average numbers of *o*-BMVC foci per nucleus in thyroid samples were approximately 16 for positive cases and 2.7 for negative cases with reference to pathological reports. It appears that the average numbers of *o*-BMVC foci are different for different types of cancer cells but less than 5 for most normal cells. In addition, similar threshold values of 22 and 20 were obtained from their ROC curves. Thus, the algorithm proposed for data analysis to diagnose FNA smears of thyroid nodules may also be valid for diagnosing other types of cancer cells.

In this work, the 95.6% consistency rate of the *o*-BMVC test to FNA cytology for both determined cases suggests that the *o*-BMVC test is similar to FNA cytology for diagnosing thyroid nodules. However, they are quite different in several characteristics. FNA cytology is a subjective method based on cellular morphology, while the *o*-BMVC test is an objective method based on the digital number of *o*-BMVC foci. For satisfactory FNA smears, the *o*-BMVC test yields only two results, either negative for benign cases or positive for malignant cases, while FNA cytology has more categories including atypia, follicular neoplasm, and suspicious as indeterminate cases. The former provides unambiguous information for clinical decision making, while the latter may provide ambiguous information. Of importance is that the *o*-BMVC test can discriminate 75.0% (60/80) of cytologically nondiagnostic cases and 92.3% (24/26) of cytologically indeterminate cases into benign or malignant cases.

In addition, the number of nondiagnostic cases from the *o*-BMVC test was markedly less than that from cytological examination. Moreover, recent study by minimizing the contribution from small size of FNA nodules in the images could markedly reduce the number of nondiagnostic case. In this work, fine-needle aspirates were first used for cytological examination, and residual aspirates were then used for the *o*-BMVC test. Considering both nondiagnostic cases reported from these two methods, it is possible that the number of nondiagnostic cases can be further reduced to 20 cases if sufficient samples were used for the *o*-BMVC test. Such possible decrease from 80 nondiagnostic cases to near 20 cases by using the *o*-BMVC test could diminish diagnostic uncertainty and benefit clinical decision making, which can reduce pending cases and health care costs.

In comparison with the 42 pathological results, the limited data gave a diagnostic accuracy of 90% (18/20) for the 20 cytologically determined cases with 2 false-negative cases. For these 20 cases, the *o*-BMVC test gave a diagnostic accuracy of 94.1% (16/17) with 1 false-negative cases and 3 nondiagnostic cases. For the 22 cytologically undetermined cases, the *o*-BMVC test showed 9 positive cases, 12 negative cases, and 1 nondiagnostic case. The diagnostic accuracy of the *o*-BMVC test for the discrimination of cytologically undetermined cases was 71.4% (15/21) with 5 false-negative cases and 1 false-positive case. Notably, the false-negative reports from the *o*-BMVC test may be due to insufficient samples caused by the use of residual samples. Thus, the use of sufficient FNA samples may reduce the number of false-negative cases and improve the diagnostic accuracy.

**Figure 5:**
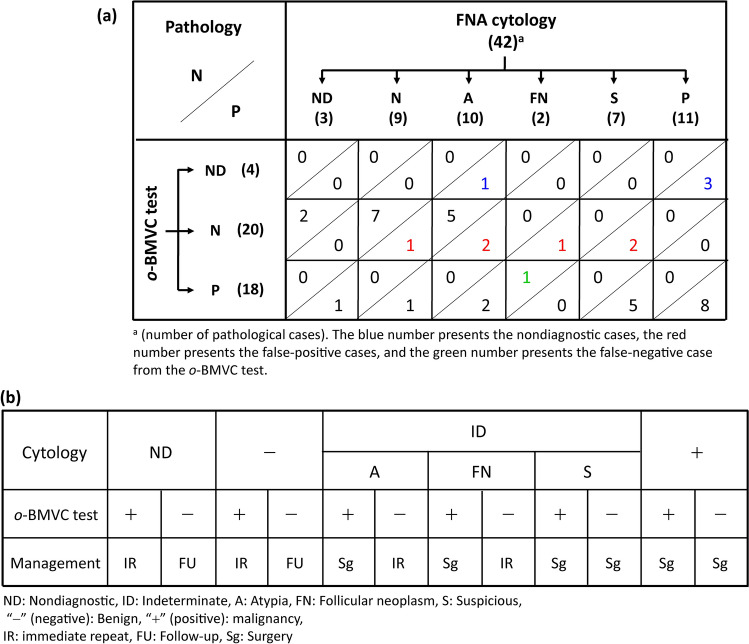
A cross correlation of pathological results to the *o*-BMVC test and cytological results **(a)**. A proposed algorithm for the management of thyroid nodules based on the combination of FNA cytology and the *o*-BMVC test **(b)**.

To elucidate whether the *o*-BMVC test can benefit clinical decision making, a cross correlation of pathological results to the *o*-BMVC test and cytological results is shown in Fig. 5a. The *o*-BMVC test discriminated 60 cytologically nondiagnostic cases into 57 benign and 3 malignant cases. Notably, a limitation of this study is that most specimens lack pathological results. Here, only three cytologically nondiagnostic cases had pathological reports. The two negative and one positive cases determined by the *o*-BMVC test are all consistent with their pathological results. For the nine cytologically benign cases, the *o*-BMVC test reported seven negative and one positive cases together with one false-negative case from both the *o*-BMVC test and cytological examination. The limited data of two positive cases suggest that the *o*-BMVC test can reduce the risk of missing cancers. For the ten cytologically atypia cases, the pathological reports showed five negative and five positive cases, while the *o*-BMVC test showed one nondiagnostic, five negative, two positive, and two false-negative cases. It is suggested that the atypia cases with negative results of the *o*-BMVC test deserve repeated examination before surgical treatment. The two atypia cases with positive results from both the *o*-BMVC test and pathological reports suggest that such cases can be treated as malignant cases.

However, the *o*-BMVC test failed to diagnose two cytologically follicular neoplasm cases, including one false-negative and one false-positive. Apparently, the use of *o*-BMVC test to examine follicular neoplasm cases deserves more study to elucidate whether such fail is due to simply incorrect diagnosis or any other reason. For the seven cytologically suspicious cases, they were all pathologically positive, while the *o*-BMVC test reported five positive and two false-negative cases. The 11 cytologically malignant cases were all pathologically positive, while the *o*-BMVC test reported eight positive and three nondiagnostic cases. In practice, the FNA cytology is a well-established method and the *o*-BMVC test is a newly developed method for the diagnosis of thyroid nodules. Thus, the decision making for the cytologically suspicious and malignant cases may be treated similarly. Accordingly, an algorithm based on the combination of the *o*-BMVC test and FNA cytology is summarized for the better management of thyroid nodules (Fig. 5b).

The major limitation of this pilot study is the small sample size of FNA nodules used for the *o*-BMVC test in a single center investigation, particularly regarding the limited pathological results. Thus, a large cohort of patients is needed to ensure uniqueness of the *o*-BMVC test for its extensive use in health care. Further study to establish the databank and optimize the performance can facilitate the use of the *o*-BMVC test. For example, the addition of artificial intelligence (AI) and machine learning holds substantial promise in accelerating the *o*-BMVC test and improving its diagnostic accuracy. It is likely that with the development of such methods, dealing with a digital number of *o*-BMVC tests should be easier than determining the morphology of FNA cytology, and these methods should be established for the management of large amounts of images.

In summary, we demonstrate that the *o*-BMVC test provides an objective method for imaging diagnosis of FNA nodules of thyroid cancer based on the digital number of *o*-BMVC foci in the nucleus. The *o*-BMVC test can markedly decrease the number of nondiagnostic cytology, discriminate indeterminate cytology into benign and malignant cases, and reduce the risk of missing cancers. Thus, the unambiguous information provided by the *o*-BMVC test for clinical decision making could reduce unnecessary costs and surgeries. This pilot study strongly suggests that the *o*-BMVC test holds great promise as an adjuvant method for the diagnosis of thyroid nodules and probably other cancers.

## Methods

### Chemical properties of *o*-BMVC molecule

The synthesis of *o*-BMVC can be found elsewhere^25^. This molecule barely fluoresces in buffer but strongly fluoresces upon interaction with G4 structures by nearly two orders of magnitude.

### Study cohort

Of the 318 patients with 327 FNAs, 271 patients were female, with a median age of 56.1 years (range, 18–88 years). The patients were enrolled between October 2018 and November 2019 in National Taiwan University Hospital. In addition, we collected 9 FNA samples for the follow-up examination from May 2020 to December 2020. The study protocol was reviewed and approved by the Institutional Review Board of National Taiwan University Hospital. All patients provided written informed consent prior to participating in the study.

### Patient sample preparation for cytological examination and the *o*-BMVC test

A 22-gauge needle was introduced into the thyroid nodules. The major aspirates were directly used as smears. After the smears were air-dried, Riu staining (a modified Romanowsky stain, Muto Pure Chemicals, Tokyo, Japan) and Papanicolaou staining were performed on all smears for cytological examination. The residual aspirates in the needle and syringe were washed with 200 μL of phosphate buffered saline (PBS). Cellular materials were centrifuged for 5 min (250 × *g*). Each residual sample was used to prepare at least two FNA smears for the *o*-BMVC test. The residual cells were fixed with 70% ethanol on microscope slides for 10 min and then stained with 5 μM of *o*-BMVC for 10 min at room temperature for the FLIM test. Notably, the results from the *o*-BMVC test for FNA smears of thyroid nodules were obtained independently from cytological examination and then compared to the cytological and pathological results.

### Fluorescence lifetime imaging microscopy (FLIM)

The setup of the FLIM system consisted of a picosecond diode laser (laser power, 5 mW) with an emission wavelength of 470 nm (LDH470; PicoQuant, Germany) and a ~ 70 ps pulse width for the excitation of *o*-BMVC under a scanning microscope (IX-71 and FV-300; Olympus, Japan). The fluorescent signal from *o*-BMVC was collected using a 60 × NA = 1.42 oil-immersion objective (PlanApoN; Olympus, Japan), passed through a 550/88 nm bandpass filter (Semrock, USA), and detected using a single-photon avalanche diode (SPAD) (PD-100-CTC; Micro Photon Devices, Italy). The fluorescence lifetime was recorded and analyzed using a time-correlated single-photon counting (TCSPC) module and software (PicoHarp 300 and SymPhoTime v5.3.2; PicoQuant, Germany). FLIM images were constructed from pixel-by-pixel lifetime information.

### Quantitative analysis of *o*-BMVC foci

Since the fluorescent decay time is longer upon binding to G4s than other structures, the acquired FLIM results of *o*-BMVC to map the G4s were presented in pseudocolor and separated into two channels: white (decay time ≥ 2.4 ns) and red (decay time < 2.4 ns). The details of quantitative analysis of *o*-BMVC foci can be found elsewhere^23,26^. Briefly, the use of the Otsu threshold method was to eliminate the weaker signals, which may be due to the loose binding of G4 DNA or the non-specific binding of small cell fragments, in the longer lifetime channel. The Otsu threshold method^30^ was used to find an optimal threshold (*T*_*opt*_) to separate two clusters or the mixture of Gaussians, with the following formula:

$$ T_{opt} = \arg \max \left\{ {\frac{{P(T)[1 - P(T)][m_{f} (T) - m_{b} (T)]^{2} }}{{P(T)\sigma_{b}^{2} (T) + [1 - P(T)]\sigma_{f}^{2} (T)}}} \right\} $$

where *P(T)* is the cumulative probability, *m*_*b*_* (T)* is the mean of the background, *m*_*f*_* (T)* is the mean of the foreground, *σ*_*b*_^*2*^*(T)* is the variance of the background and*σ*_*f*_^*2*^*(T)* is the variance of the foreground. After applying the Otsu threshold method, the weak signals can be eliminated, while the stronger signals can be preserved. Such image analysis allows us to lower the possible counting errors in human eye detection and unambiguously quantify the number of foci in different cell lines.

### Ethical approval

All the experiment protocol for involving humans was in accordance to international guidelines and the Declaration of Helsinki. This study was approved by the Ethic Committee of the National Taiwan University Hospital (NTUH) (IRB No. 201411011RINC).

## Supplementary Information

Supplementary Information.
