# Supplementary material for: Pilot imaging study of o-BMVC foci for discrimination of indeterminate cytology in diagnosing fine-needle aspiration of thyroid nodules
Source: Sci Rep. 2021 Dec 6;11:23475. doi: 10.1038/s41598-021-02887-8 (PMC8648827; doi:10.1038/s41598-021-02887-8)
Supplement: Supplementary file 1 — Supplementary Information. [file 41598_2021_2887_MOESM1_ESM.pdf]

Table S1. The results of FNA cytology, the *o*-BMVC test, and the pathology of the 327 thyroid nodules.

Number	Cytology results	Pathology results	*o*-BMVC		Sex	Age	Cytology report	Pathology report
			Results	S				
1	A	N	N	2	F	57	A few atypical follicular cells with pallor chromatin, grooving, and mild irregular nuclear membrane are present.	Follicular adenoma
2	N	--	N	0	F	38	cells with macrophages and colloid	
3	ND	--	N	0	F	75	Blood only	
4	N	--	N	0	F	56	A few sheets of follicular cells and colloid	
5	P	P	P	6	F	39	Papillary thyroid carcinoma	Papillary thyroid cancer
6	N	--	N	0	F	73	Clusters of follicular cells and Hurthle cells with abundant colloid	
7	N	--	N	0	F	39	Sheets of follicular cells, macrophages and colloid	
8	N	--	N	0	F	51	Small clusters of Hurthle cells with a few macrophages and colloid	
9	FN	P	N	0	F	32	Low cellularity. Small clusters of follicular cells with colloid	Papillary thyroid cancer
10	N	--	N	0	M	65	Few follicular cells with colloid	
11	ND	--	N	0	F	70	Macrophages and colloid	

12	ND	--	N	0	F	61	Insufficient cellularity for interpretation, few Hurthle cells, macrophages and colloid	
13	N	--	N	0	M	40	A few sheets of follicular cells, macrophages and colloid	
14	A	P	P	8	F	61	Atypical histiocytoid cells and macrophages are present in the fluid background. Please take a biopsy, if clinically indicated.	Papillary thyroid cancer
15	S	P	P	9	F	55	Suspicious for metastatic papillary carcinoma. Scanty cellularity smears show a few atypical cells with enlarged nuclei, intranuclear pseudoinclusions, nuclear membrane irregularity, and nuclear molding, but no lymphoid components are seen. Please verify the aspiration site.	Papillary thyroid cancer
16	N	P	P	6	F	51	Few sheets of follicular cells with macrophages and colloid	Papillary thyroid cancer
17	S	P	P	8	F	54	Suspicious for papillary carcinoma, clusters of enlarged atypical cells with nuclear grooves and rare intranuclear pseudoinclusions	Papillary thyroid cancer
18	S	P	P	7	F	51	Suspicious for papillary carcinoma, clusters of enlarged atypical cells	Papillary thyroid cancer

							with nuclear grooves and rare intranuclear pseudoinclusions	
19	N	--	N	0	F	55	A few clusters of follicular cells with colloid	
20	N	--	N	0	F	38	Small clusters of follicular cells with colloid	
21	P	P	P	9	F	82	Papillary carcinoma. Monolayer sheets of atypical cells show cellular enlargement, nuclear membrane irregularity, nuclear molding, nuclear grooves, and intranuclear pseudoinclusions. Multinucleated giant cells and thick colloid are present.	Papillary thyroid cancer
22	ND	--	N	2	F	57	Few follicular cells, macrophages and colloid	
23	N	--	N	0	F	64	Sheets of follicular cells and colloid	
24	N	--	N	0	F	42	Low cellularity, a few follicular cells, old RBCs and colloid	
25	N	--	N	0	F	64	Sheets of follicular cells with colloid, R	
26	N	--	N	0	F	62	A few sheets of follicular cells and colloid	
27	N	--	N	1	F	71	Sheets of follicular cells with colloid, R	

28	N	--	N	1	F	59	Low cellularity. A few small clusters of follicular cells with colloid	
29	N	--	N	0	F	66	Sheets of follicular cells, old RBCs and colloid	
30	N	--	N	0	F	71	Sheets of follicular cells with macrophages and colloid, R	
31	ND	--	N	0	M	56	Few small clusters of follicular cells with colloid, R	
32	N	--	N	0	M	64	A few sheets of follicular cells with macrophages and colloid	
33	N	--	N	0	M	66	Sheets of follicular cells, macrophages, cholesterol crystals and colloid	
34	N	--	N	0	F	71	A few clusters of follicular cells with abundant colloid	
35	N	--	N	0	M	66	Small clusters of follicular cells and Hurthle cells with a few macrophages and colloid	
36	ND	--	N	0	F	60	Naked nuclei and colloid	
37	ND	P	P	9	M	68	Few naked nuclei and colloid	Papillary thyroid cancer
38	N	N	N	0	F	68	Scant follicular cells and colloid	nodular hyperplasia
39	N	--	N	0	F	64	A few follicular cells with colloid	
40	N	--	N	0	F	42	A few follicular cells with colloid	

41	N	--	N	2	F	63	Sheets of follicular cells with macrophages and colloid	
42	A	--	N	1	F	68	Mixed population of lymphoid cells. Please verify the aspiration site.	
43	N	N	N	0	F	50	Negative for malignant cells	Nodular hyperplasia
44	N	--	N	0	M	69	A few clusters of follicular cells with colloi	
45	A	P	P	6	F	39	Low cellularity, few clusters of atypical follicular cells with enlarged nuclei are present with colloid.	Papillary thyroid cancer
46	N	--	N	1	F	41	A few follicular cells, blood and colloid	
47	P	P	P	8	F	48	Papillary carcinoma	Papillary thyroid cancer
48	FN	N	P	7	F	64	Sheets of atypical follicular cells are admixed with scant colloid. The atypical cells show enlarged nuclei and microfollicular arrangements. Follicular neoplasm is suspected.	Adenomatous hyperplasia
49	N	--	N	0	F	48	Clusters of follicular cells with colloid, R	
50	ND	--	N	0	F	69	Few clusters of follicular cells with colloid, R	
51	N	--	N	1	F	50	Sheets of follicular cells, macrophagess and colloid	

52	ND	--	N	0	F	65	Macrophages, lymphocytes, old RBCs and colloid	
53	ND	--	N	1	F	29	Blood cells only	
54	N	--	N	0	F	47	Few sheets of follicular cells with colloid	
55	ND	--	N	0	F	67	Blood cells only	
56	N	--	N	0	F	42	A few small cluster of follicular cells with colloid	
57	N	--	N	0	F	35	A few clusters of follicular cells with increased lymphoid cells and colloid.	
58	N	--	N	2	F	64	A few clusters of follicular cells with colloid.	
59	N	--	P	9	F	69	Clusters of follicular cells, macrophages and colloid, L.	
60	ND	N	N	0	M	65	Macrophages, old RBCs and colloid.	Nodular hyperplasia
61	N	--	N	0	F	50	A few clusters of follicular cells and Hurthle cells with colloid.	
62	N	--	P	9	F	72	A few sheets of follicular cells and colloid.	
63	N	--	N	0	F	69	Sheets of follicular cells with colloid, R	
64	ND	--	N	1	F	33	Blood and colloid	
65	N	--	P	9	F	46	Clusters of follicular cells and colloid	
66	N	--	N	1	F	27	Sheets of follicular cells and Hurthle cells, macrophages and colloid.	

67	ND	--	N	0	F	53	Colloid only	
68	N	--	P	9	F	52	Sheets of follicular cells with macrophages and colloid.	
69	A	--	N	0	M	65	Few atypical follicular cells with enlarged nuclei and irregular nuclear contours.	
70	N	--	P	8	F	28	Few sheets of follicular cells with macrophages and colloid	
71	N	--	P	8	F	75	Small clusters of follicular cells with a few macrophages and colloid	
72	P	P	P	8	F	75	Papillary carcinoma, clusters of enlarged cells with nuclear grooves and intranuclear pseudoinclusions.	Papillary thyroid cancer
73	N	--	N	0	F	53	Clusters of follicular cells and Hurthle cells with macrophages and colloid.	
74	ND	--	N	0	F	41	Few degenerated cells with macrophages are present, R.	
75	P	--	P	8	F	60	Papillary thyroid carcinoma, L	
76	N	--	N	0	F	72	A few clusters of follicular cells with mixed population of lymphoid cells and colloid.	
77	N	--	N	0	F	67	Sheets of follicular cells with macrophages and colloid.	
78	N	--	N	0	F	42	Sheets of follicular cells with colloid, R	

79	N	--	N	0	F	58	Sheets of follicular cells with macrophages and colloid	
80	A	P	N	0	F	75	Sheets of atypical follicular cells and scant colloid are present. The atypical cells show enlarged nuclei with irregular contours and anisonucleosis.	Papillary thyroid cancer
81	ND	--	N	0	F	41	Insufficient cellularity for interpretation, macrophages and colloid, R	
82	N	--	N	0	F	25	A few sheets of follicular cells, macrophages and colloid	
83	N	--	N	0	M	22	Follicular cells are present, R	
84	A	--	N	0	F	56	A few atypical follicular cells with enlarged, crowded nuclei with mildly irregular nuclear membrane are present. R.	
85	ND	--	N	0	F	60	A few naked nuclei with blood and colloid, R	
86	N	--	N	0	F	74	Hashimoto's thyroiditis, L	
87	A	N	N	0	F	53	Few atypical histiocytoid cells admixed with follicular cells and colloid, L.	Nodular hyperplasia
88	ND	--	N	0	M	66	Blood cells and colloid, Isthmus	
89	P	P	P	9	F	54	Medullary carcinoma, R.: atypical cells with granular cytoplasm and	Medullary carcinoma

							eccentric nuclei.	
90	N	--	N	0	F	67	Few sheets of follicular cells with colloid,	
91	ND	--	N	0	F	49	Few follicular cells and colloid, R	
92	N	--	N	0	F	53	A few sheets of follicular with colloid, R	
93	ND	--	N	0	F	48	Blood cells and colloid, R	
94	S	P	P	9	F	29	Supicious for papillary carcinoma. Clusters of suspicious cells with frequent nuclear grooves but rare intranuclear cytoplasmic invagination are present, L	Papillary thyroid cancer
95	N	--	N	0	F	51	A few sheets of follicular with colloid, R	
96	N	--	N	0	F	45	Sheets of follicular cells with colloid, L	
97	N	--	N	0	F	60	Few sheets of follicular cells with macrophages and colloid, isthmus	
98	S	P	N	0	M	65	Suspicious for papillary thyroid carcinoma, R. It shows follicular cells with enlarged, crowded nuclei with pallor chromatin, and irregular nuclear membrane.	Papillary thyroid cancer
99	A	N	N	0	F	64	A few atypical follicular cells with microfollicles are present, R.	Bilateral nodular hyperplasia

100	N	--	N	0	F	40	Sheets of follicular cells and Hurthle cells with colloid, R	
101	ND	--	P	7	F	61	Macrophages , old RBCs and colloid, R	
102	N	--	N	0	M	64	A few degenerated follicular cells with colloid. R	
103	ND	--	N	0	F	63	Few lymphoid cells with blood, L	
104	N	--	N	0	F	46	A few sheets of follicular cells with macrophages and colloid, L	
105	N	--	N	0	F	68	Sheets of follicular cells and colloid	
106	N	--	N	0	F	44	A few follicular cells and colloid	
107	ND	--	N	0	F	49	Blood cells, L	
108	N	--	N	0	F	73	A few sheets of follicular cells and colloid, L	
109	N	--	N	0	M	46	A few follicular cells with macrophages, old RBCs and colloid, R	
110	N	--	N	0	F	50	Sheets of follicular cells, macrophages and colloid, L	
111	N	--	N	0	F	54	Sheeets of Hurthle cells and follicular cells with lymphocytes and colloid, R.	
112	ND	--	N	0	F	56	Mixed population of lymphoid cells, L	
113	N	--	N	0	F	37	Sheets of follicular cells and Hurthle	

							cells with a few immature lymphoid cells and colloid, consistent with autoimmune thyroiditis R	
114	ND	--	N	0	M	57	Blood cells, L	
115	N	N	N	0	F	66	A few small clusters of follicular cells and colloid, R	Nodular hyperplasia
116	N	--	N	0	F	33	Sheets of follicular cells with colloid, L and R	
117	N	N	N	0	F	43	Few sheets of follicualr cells with colloid, R	Nodular hyperplasia
118	N	--	N	0	M	76	Few sheets of follicular cells with colloid, R	
119	ND	--	N	0	M	55	Few naked nuclei and blood, R	
120	N	--	N	0	F	69	Few sheets of follicular cells with macrophages and colloid, R	
121	N	--	N	0	F	65	few sheets of follicular cells and colloid, isthmus	
122	ND	--	N	0	F	53	Few follicular cells, macrophages, blood cells and colloid. L	
123	N	--	N	0	F	64	Sheets of follicular cells with colloid, isthmus	
124	N	P	N	0	M	68	Sheets of follicular cells, macrophages, old RBCs and colloid, R	Papillary thyroid cancer
125	ND	--	N	0	F	69	Blood and colloid, R	

126	N	--	N	0	M	65	A few sheets of follicular cells and colloid, L	
127	N	--	N	0	F	83	Sheets of follicular cells, macrophages and colloid, R	
128	N	--	N	0	F	62	Clusters of follicular cells with a few macrophages and colloid, L	
129	N	--	N	0	F	65	Clusters of follicular cells with a few macrophages and colloid, R	
130	N	--	N	0	F	63	Small clusters of follicular cells with few macrophages and colloid, R	
131	ND	--	N	0	F	55	Mixed population of lymphoid cells. There is no follicular cell or Hurthle cell. R.	
132	N	--	N	0	F	62	Low cellularity. Small clusters of follicular cells with macrophages and colloid, L	
133	ND	--	N	0	F	69	Few follicular cells and Hurthle cells with colloid, L	
134	P	P	P	7	F	18	Papillary thyroid carcinoma, L	Papillary thyroid cancer
135	ND	--	N	0	F	57	A few naked nuclei, blood and colloid, L	
136	N	--	N	0	F	45	A few follicular cells with macrophages and colloid. R	
137	S	P	N	2	F	47	Suspicious for papillary carcinoma, R.: a few clusters of atypical cells	Carcinoma, c/w clear cell carcinoma

							with nuclear membrane irregularity, nuclear grooves and intranuclear pseudoinclusions	
138	ND	--	N	0	F	31	Macrophages, cholesterol crystals and colloid, L	
139	ND	--	N	1	F	48	Few follicular cells and blood, L	
140	ND	--	N	0	F	51	Few clusters of follicular cells with colloid, L	
141	N	--	N	0	M	57	Clusters of follicular cells with colloid, R	
142	N	--	N	0	F	61	Clusters of follicular cells with colloid, L	
143	ND	--	N	0	F	67	Blood cells, L	
144	N	--	N	0	F	36	Sheets of follicular cells, macrophages and colloid, L	
145	P	P	P	4	F	46	Carcinoma. Medullary carcinoma or other metastatic neuroendocrine carcinoma is considered. Please correlate with clinical status. R	Medullary carcinoma
146	N	--	N	0	M	55	Sheets of follicular cells, macrophages and colloid, isthmus	
147	A	P	N	0	F	41	A few sheets of atypical follicular cells are admixed with small follicular cells and colloid. The atypical cells show enlarged nuclei with irregular contours and pale	Papillary thyroid cancer

							chromatin. Please re-aspirate, if clinically indicated. R	
148	ND	--	N	0	F	26	Few naked nuclei and blood, R	
149	N	--	N	2	F	38	Sheets of follicular cells and colloid, L	
150	A	--	N	0	F	31	It shows clusters of atypical follicular cells with enlarged nuclei, with mildly irregular nuclear membrane. Multinucleated giant cells and colloid are also noted, R	
151	N	--	N	0	M	65	A few clusters of follicular cells and colloid, L	
152	N	--	N	0	F	53	Sheets of follicular cells, macrophages and colloid, R	
153	N	--	N	0	F	58	A few sheets of follicular cells with macrophages and colloid, R	
154	N	N	N	1	F	41	Sheets of follicular cells with colloid, R	Nodular hyperplasia
155	N	--	N	0	F	57	Few sheets of follicular cells with colloid, L	
156	N	--	N	1	F	63	Few sheets of follicular cells with colloid, R	
157	N	--	N	0	F	62	Abundant colloid and few follicular cells, L	
158	ND	--	N	0	M	68	Blood cells, L	
159	N	--	N	0	F	62	Sheets of follicular cells and Hurthle	

							cells with colloid, L	
160	N	--	N	1	F	58	Few sheets of follicular cells with colloid, R	
161	N	--	N	0	M	55	Few sheets of follicular cells with colloid, R	
162	N	--	N	0	M	45	Sheets of follicular cells with colloid. L	
163	N	--	N	0	F	59	Some degenerated Hurthle cells with blood and colloid. R	
164	N	--	N	0	F	37	A few follicular cells with macrophages, cholesterol crystals and colloid. R	
165	N	--	N	0	M	58	Clusters of follicular cells with macrophages and colloid. L	
166	ND	N	N	0	F	49	Macrophages, a few multinucleated cells and colloid, L	Atypical adenomatous hyperplasia
167	N	--	N	0	F	47	Sheets of follicular cells and colloid, R	
168	N	--	N	0	M	61	A few follicular cells with colloid. L	
169	N	--	N	1	M	37	Sheets of follicular cells with colloid. R	
170	ND	--	N	0	F	59	Few degenerated cells with macrophages and colloid. L	
171	N	--	N	1	F	69	Clusters of follicular cells and Hurthle cells with colloid, R	

172	ND	--	N	0	F	58	Few small clusters of follicular cells with colloid, isthmus	
173	N	N	N	0	F	63	Clusters of Hurthle cells and follicular cells with colloid	Bilateral nodular hyperplasia
174	N	--	N	0	F	47	Small clusters of follicular cells are present with increased lymphoid cells, R.	
175	N	--	N	0	F	60	Clusters of follicular cells with macrophages and colloid	
176	N	--	N	0	F	63	Clusters of follicular cells, old RBCs and colloid, L	
177	N	--	N	0	F	33	Sheets of Hurthle cells and follicular cells, macrophage and colloid, L	
178	N	--	N	0	F	62	Clusters of follicular cells with macrophages and colloid, L	
179	N	--	N	0	F	59	Clustered and scattered follicular cells with colloid	
180	N	--	N	0	F	37	Clusters of follicular cells with macrophages and colloid	
181	N	--	N	0	F	63	Sheets of follicular cells and colloid, R	
182	N	--	N	0	F	70	A few sheets of follicular cells, macrophages and colloid, R	
183	N	--	N	0	F	76	Few sheets of follicular cells with colloid. L	
184	N	--	N	0	F	63	Sheets of follicular cells and colloid,	

							L	
185	N	--	N	1	F	72	Sheets of follicular cells and colloid, R	
186	N	--	N	0	F	62	A few sheets of follicular cells and colloid, L	
187	N	--	N	0	F	62	A few sheets of degenerated follicular cells and colloid, L	
188	N	--	N	0	F	53	A few sheets of follicular cells and colloid, isthmus	
189	ND	--	N	0	F	67	Blood only, R	
190	N	--	N	0	F	62	Scant follicular cells and colloid, L	
191	N	--	N	0	F	53	Scant follicular cells and colloid, R	
192	N	--	N	0	F	62	Clusters of follicular cells, macrophages and colloid, R	
193	ND	--	N	0	F	60	Mixed population of lymphoid cells, L	
194	ND	--	N	0	F	54	Few clusters of follicular cells with macrophages and colloid, L	
195	N	--	N	0	F	61	Clusters of follicular cells and colloid, R	
196	A	--	N	0	F	57	Few atypical histiocytoid cells admixed with small follicular cells, macrophages and colloid, L	
197	ND	--	N	0	F	64	Few follicular cells, blood cells and colloid, R	

198	N	--	N	0	F	68	Some clusters of follicular cells with colloid. L	
199	N	--	N	0	F	36	Small clusters of follicular cells with colloid. L	
200	N	--	N	0	F	40	Scant follicular cells and colloid, R	
201	N	--	N	0	F	65	Sheets of follicular cells and colloid, L	
202	N	--	N	0	F	50	Sheets of follicular cells, a few lymphoid cells and colloid, L	
203	N	--	N	2	F	54	A few sheets of Hurthle cells and follicular cells, macrophages and colloid, L	
204	ND	--	N	1	F	66	Blood and colloid, L	
205	N	--	N	0	F	44	Few sheets of follicular cells with colloid, R	
206	N	--	N	0	F	51	A few sheets of follicular cells with macrophages and colloid, R	
207	N	--	N	0	F	31	Sheets of Hurthle cells and follicular cells, increased lymphocytes and colloid, R	
208	N	--	N	0	F	78	Sheets of follicular cells and Hurthle cells with colloid, R	
209	N	--	N	0	F	51	Clusters of follicular cells with epithelioid cells, multinucleated giant cells and colloid, L	
210	ND	--	N	1	F	66	Bloody smears, L.	

211	ND	--	N	0	M	54	Blood and colloid. L	
212	N	--	N	1	F	70	Clusters of follicular cells with colloid. R	
213	N	--	N	0	F	78	A few small clusters of follicular cells, Hurthle cells and multinucleated giant cells with colloid, isthmus	
214	ND	--	N	0	F	30	Naked nuclei, macrophages, cholesterol crystals and colloid. L	
215	N	--	N	0	M	57	Sheets of follicular cells and Hurthle cells with colloid. L	
216	N	--	N	0	F	57	Sheets of follicular cells with increased lymphocytes and colloid. L	
217	N	--	N	0	F	52	Clusters of follicular cells, macrophages and colloid, isthmus	
218	N	--	N	0	F	60	Clusters of follicular cells, macrophages and colloid, R	
219	ND	--	N	1	F	43	Few clusters of follicular cells, multinucleated cells and colloid, L	
220	N	--	N	0	F	56	Clusters of follicular cells and colloid, R	
221	N	--	N	0	F	38	A few clusters of follicular cells, a few Hurthle cells, epithelioid cells, increased lymphoid cells and colloid are present. R	
222	N	--	N	0	F	47	A few sheets of follicular cells with colloid, R	

223	N	--	N	0	F	61	Clusters of follicular cells with colloid. L	
224	N	--	N	0	M	63	Sheets of follicular cells with colloid. R	
225	ND	--	N	0	M	46	Naked nuclei and colloid. R	
226	N	--	N	1	F	33	Scattered follicular cells with colloid. L	
227	N	--	N	0	F	54	A few loose clusters of follicular cells and colloid, isthmus	
228	ND	--	N	0	F	67	Macrophages, old RBCs and colloid, L	
229	N	--	N	0	F	49	Sheets of follicular cells and Hurthle cells with colloid, L	
230	N	N	N	0	F	56	Scattered and sheeted follicular cells with colloid, L	Nodular hyperplasia
231	ND	--	N	0	F	43	Naked nuclei and colloid, L	
232	N	--	P	9	F	44	Sheets of follicular cells with colloid, R	
233	N	--	N	0	F	60	Sheets of follicular cells with colloid, R	
234	N	--	N	2	F	53	Sheets of follicular cells and colloid, L	
235	ND	--	N	1	F	69	Few naked nuclei and colloid, L	
236	ND	--	N	0	F	62	Colloid, R	

237	N	--	N	0	F	59	Sheets of follicular cells, macrophages and colloid, R	
238	A	N	N	0	F	44	A few sheets of atypical follicular cells with enlarged nuclei and anisonucleosis admixed with follicular cell sheets, Hurthle cell sheets and increased lymphoid cells	Nodular goiter
239	N	--	N	0	F	66	Sheets of follicular cells with colloid. L	
240	N	--	N	1	F	65	Sheets of follicular cells with colloid, R	
241	N	--	N	0	F	82	Sheets of follicular cells with colloid, L	
242	N	--	N	0	F	88	Sheets of follicular cells and Hurthle cells with colloid, L	
243	N	--	N	0	F	69	A few sheets of follicular cells and colloid, R	
244	N	--	N	0	F	69	Sheets of follicular cells and Hurthle cells with colloid, L	
245	N	--	N	0	F	75	Sheets of follicular cells and colloid, R	
246	N	--	N	0	F	56	Sheets of follicular cells with abundant colloid, L	
247	ND	--	N	0	F	57	Blood cells and colloid, L	
248	N	--	N	1	F	66	Sheets of Hurthle cells with lymphoid cells and colloid,	

							consistent with Hashimoto's thyroiditis, L.	
249	N	--	N	0	F	53	A few follicular cells with abundant colloid, R	
250	ND	--	N	0	F	63	A macrophage with blood cells and colloid, L	
251	N	--	N	0	F	62	Sheets of follicular cells and Hurthle cells with macrophages and colloid, L	
252	N	--	N	0	F	54	Sheets of follicular cells, macrophages and colloid, R	
253	ND	--	N	0	F	46	A few naked nuclei, macrophages, blood cells and colloid, L	
254	N	--	N	0	M	50	Sheets of follicular cells, macrophages and colloid, R	
255	N	--	N	0	F	62	A few follicular cells with abundant colloid, R	
256	ND	--	N	0	F	81	Few naked nuclei with blood cells and colloid, L	
257	S	P	P	8	F	51	Suspicious for papillary carcinoma, incomplete nuclear changes pattern, L.: There is generalized nuclear enlargement. Nuclear grooves are evident, but nuclear molding is minimal. Intranuclear pseudoinclusions are rare.	Papillary thyroid cancer

258	A	--	N	0	F	45	A few sheets of atypical follicular cells and colloid are present. The atypical cells show microfollicular arrangements. R.	
259	P	--	P	6	M	38	Papillary carcinoma, R.: Papillary clusters and monolayer sheets of enlarged cells show nuclear grooves, nuclear membrane irregularity and intranuclear pseudoinclusions. Multinucleated giant cells, thick colloid, and atypical cells with squamoid cytoplasm are present.	
260	ND	--	N	0	M	73	Few follicular cells and blood, R	
261	ND	--	P	8	F	46	few follicular cells and blood, L	
262	A	N	N	0	F	80	A few sheets of atypical follicular cells show enlarged nuclei with irregular contours. L	Bilateral multinodular hyperplasia
263	P	--	P	9	F	71	Papillary carcinoma, L.: papillary clusters of enlarged cells with nuclear grooves and intranulcear pseudoinclusions.	
264	N	--	N	0	F	56	Sheets of follicular cells with macrophages and colloid, L	
265	P	P	P	9	M	36	Papillary thyroid carcinoma, R	Papillary thyroid cancer
266	N	--	N	0	M	58	Sheets of follicular cells with colloid,	

							R	
267	ND	--	ND	--	F	43	Insufficient cellularity for interpretation, macrophages with old RBCs	
268	ND	--	ND	--	F	66	Blood only	
269	N	--	ND	--	F	60	Clusters of follicular cells with colloid	
270	ND	--	ND	--	F	54	Insufficient cellularity for interpretation, a few follicular cells with macrophages and colloid	
271	N	--	ND	--	F	52	Low cellularity. A few small clusters of follicular cells with colloid	
272	A	P	ND	--	F	56	Sheets of atypical follicular cells with enlarged nuclei and mildly irregular nuclear membrane are present.	Papillary thyroid cancer
273	ND	--	ND	--	F	57	Few epithelial cells with macrophages, PMNs, old RBCs and colloid are present in the fluid specimen.	
274	N	--	ND	--	F	62	Scant follicular cells, macrophages and colloid	
275	A	--	ND	--	M	63	Sheets of cuboidal cells and colloid-like substance are present in the lymphoid background. The nature is indeterminate.	
276	ND	--	ND	--	F	67	Blood only	

277	P	P	ND	--	F	70	Papillary thyroid carcinoma. It shows papillary thyroid carcinoma with enlarged, crowded nuclei with irregular nuclear membrane and pallor chromatin in monolayered sheets with swirling or papillary architectures.	Papillary thyroid cancer
278	N	--	ND	--	F	65	Sheets of follicular cells and colloid	
279	N	--	ND	--	F	46	A few sheets of follicular cells, old RBCs and colloid	
280	N	--	ND	--	F	47	A few sheets of follicular cells and colloid	
281	ND	--	ND	--	F	69	Old RBCs and colloid, R	
282	N	--	ND	--	F	65	A few sheets of follicular cells with macrophages and colloid	
283	N	--	ND	--	M	66	Low cellularity. Some clusters of follicular cells with macrophages, cholesterol crystals and colloid	
284	P	P	ND	--	F	62	Papillarty carcinoma, L.:monolayer sheets of atypical cells with cellular enlargement	Papillary thyroid cancer
285	N	--	ND	--	M	58	Sheets of follicular cells with colloid, R	
286	N	--	ND	--	M	62	A few sheets of follicular cells, macrophages and colloid	

287	N	--	ND	--	M	65	Sheets of follicular cells with colloid, L	
288	N	--	ND	--	M	64	Sheets of follicular cells and colloid, R	
289	ND	--	ND	--	M	46	Blood cells and colloid, Isthmus	
290	N	--	ND	--	F	77	A few sheets of follicular cells with colloid, R	
291	N	--	ND	--	M	48	A few small clusters of follicular cells with colloid, R	
292	N	--	ND	--	F	51	Clusters of follicular cells with colloid, isthmus	
293	N	--	ND	--	F	62	Few sheets of follicular cells with colloid, R	
294	ND	--	ND	--	F	66	Blood and colloid, R	
295	ND	--	ND	--	F	49	Few clusters of degenerated follicular cells with colloid. L	
296	ND	--	ND	--	F	36	Few clusters of follicular cells with macrophages and colloid. L	
297	P	P	ND	--	F	41	Papillary carcinoma. R	Papillary thyroid cancer
298	ND	--	ND	--	F	54	Blood and colloid, R	
299	N	--	ND	--	F	68	Numerous macrophages, old RBCs and colloid, L	
300	N	--	ND	--	F	50	Scant follicular cells and colloid, R	
301	ND	--	ND	--	F	53	Numerous PMNs and lymphocytes	

							are present, no follicular cells.	
302	N	--	ND	--	F	31	Clusters of Hurthle cells with blood cells and colloid, R	
303	ND	--	ND	--	F	62	Old RBCs and colloid, R	
304	N	--	ND	--	F	33	Sheets of follicular cells with colloid, L and R	
305	N	--	ND	--	F	34	Few sheets of follicular cells with macrophages and colloid, L and R	
306	N	--	ND	--	F	34	Few sheets of follicular cells with macrophages and colloid, L and R	
307	N	--	ND	--	F	50	Sheets of follicular cells with macrophages, epithelioid cells, old RBCs and colloid, R	
308	ND	--	ND	--	F	58	A few degenerated cells, macrophages, blood cells and colloid. R	
309	N	--	ND	--	F	40	Clusters follicular cells with colloid, L	
310	ND	--	ND	--	F	44	Few follicular cells and colloid, R	
311	N	--	ND	--	F	58	Small clusters of follicular cells with colloid, R	
312	N	--	ND	--	F	56	Sheets of follicular cells and colloid, R	
313	ND	--	ND	--	F	67	Blood cells and colloid, L	
314	N	--	ND	--	F	50	A few clusters of follicular cells and colloid, R	

315	N	--	ND	--	F	68	Sheets of follicular cells, macrophages and colloid, L	
316	N	--	ND	--	F	60	A few follicular cells and colloid, L	
317	N	--	ND	--	F	74	Hashimoto's thyroiditis, R	
318	N	--	ND	--	M	47	A few sheets of follicular cells and colloid, L	
319	N	--	ND	--	F	78	Sheets of follicular cells with colloid, L	
320	N	--	ND	--	F	55	Scant follicular cells and colloid, L	
321	ND	--	ND	--	F	71	Blood and colloid, L	
322	ND	--	ND	--	M	71	Blood and colloid. L	
323	N	--	ND	--	F	44	Few degenerated cells, blood and abundant colloid. L	
324	N	--	ND	--	F	40	Clusters of follicular cells and colloid, R	
325	ND	--	ND	--	F	64	Few naked nuclei, blood and colloid, isthmus	
326	ND	--	ND	--	F	74	Few blood and colloid, R	
327	N	--	ND	--	M	57	A few follicular cells, Hurthle cells and colloid, R.	

Table S2. The results of FNA cytology and the *o*-BMVC test for these 9 repeated nodules

Number	Cytology results	*o*-BMVC results
121	N	N
	ND	N
174	N	N
	N	N
183	N	N
	N	N
194	ND	N
	ND	N
198	N	N
	N	N
266	N	N
	N	N
292	N	ND
	N	N
324	N	ND
	N	N
325	ND	ND
	N	N

Figure S1. Three images of 30 positive cases reported by the *o*-BMVC test.

#5

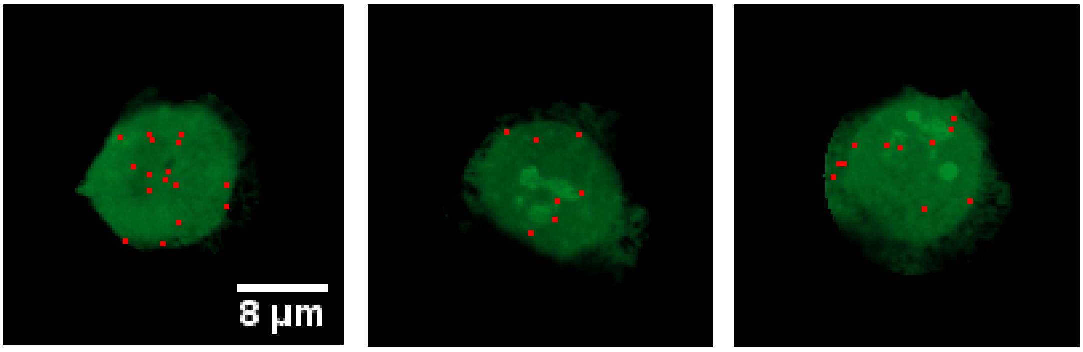

#14

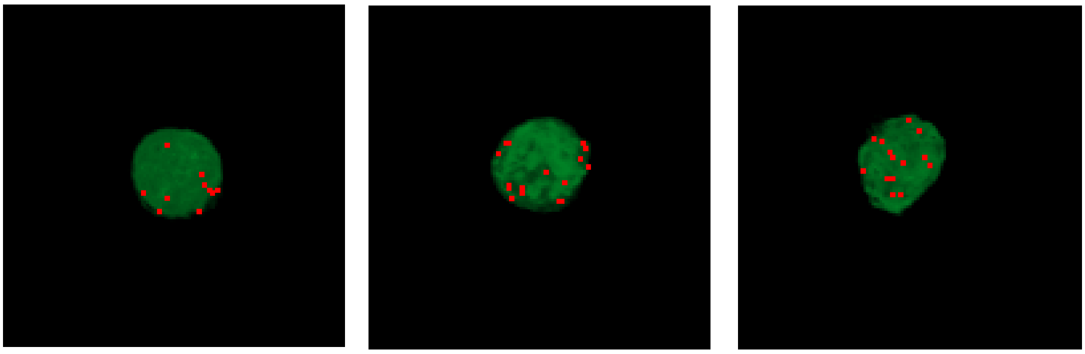

#15

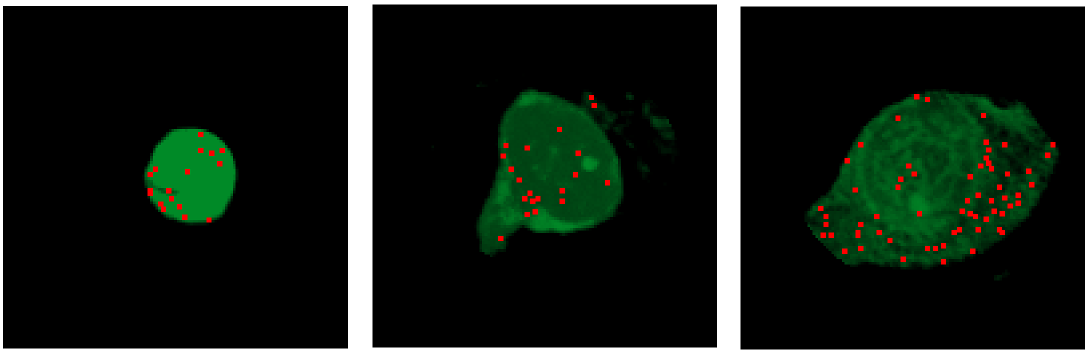

#16

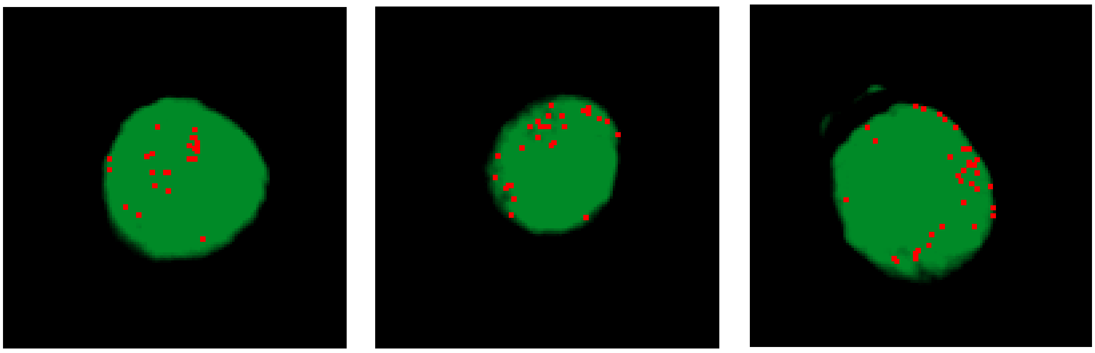

#17

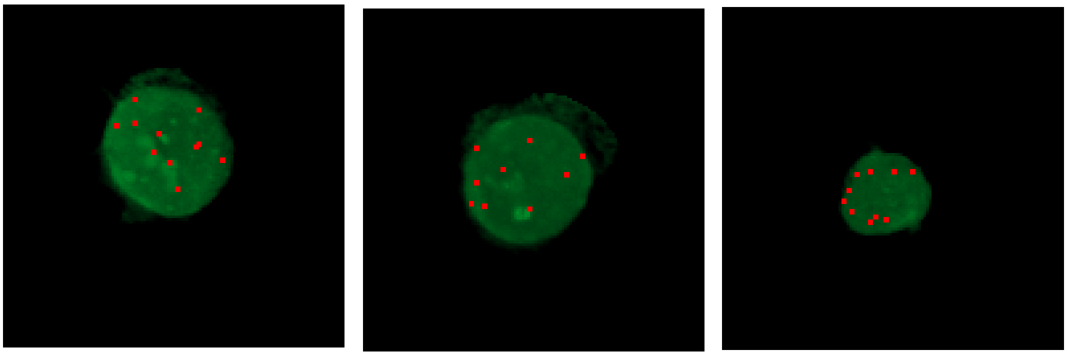

#18

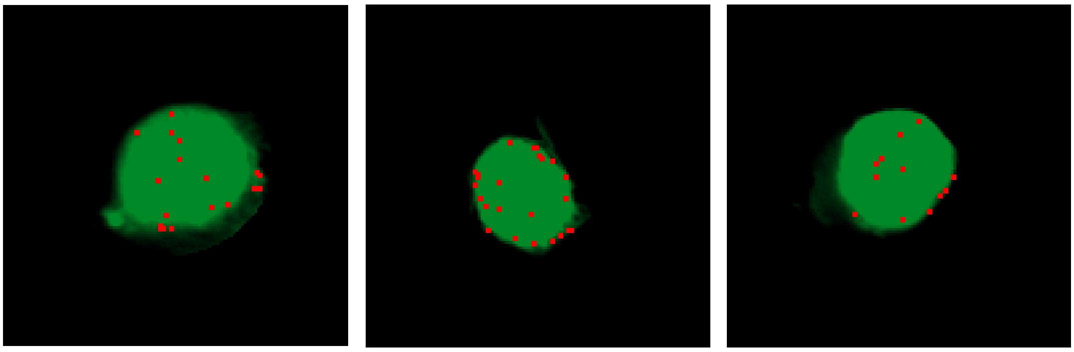

#21

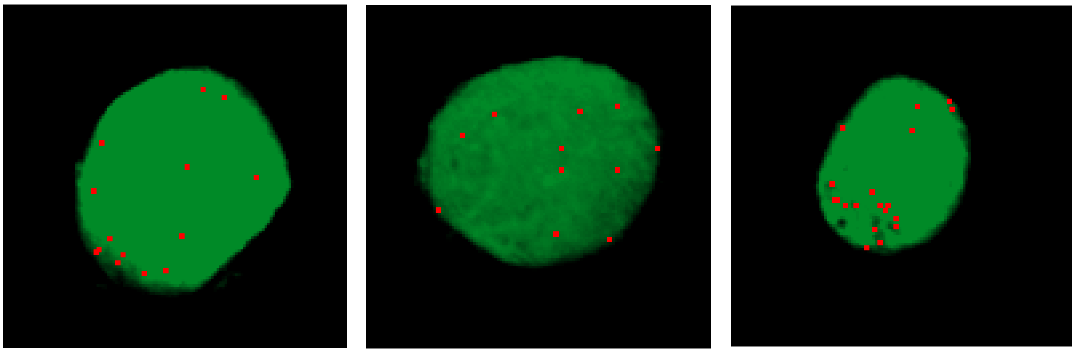

#37

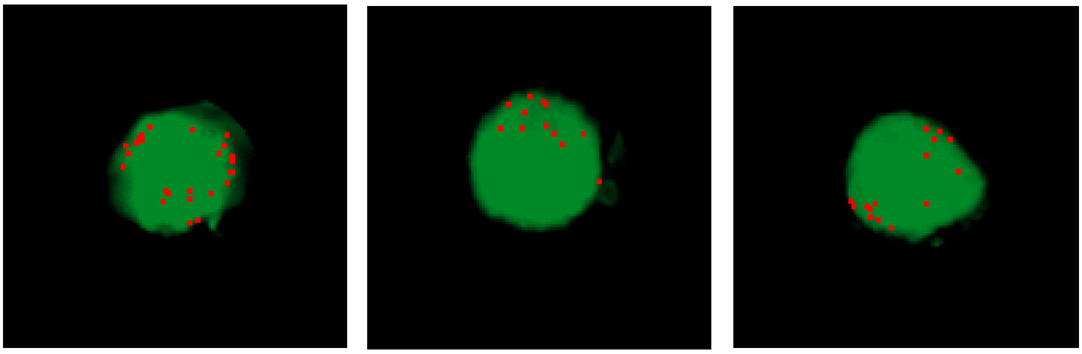

#45

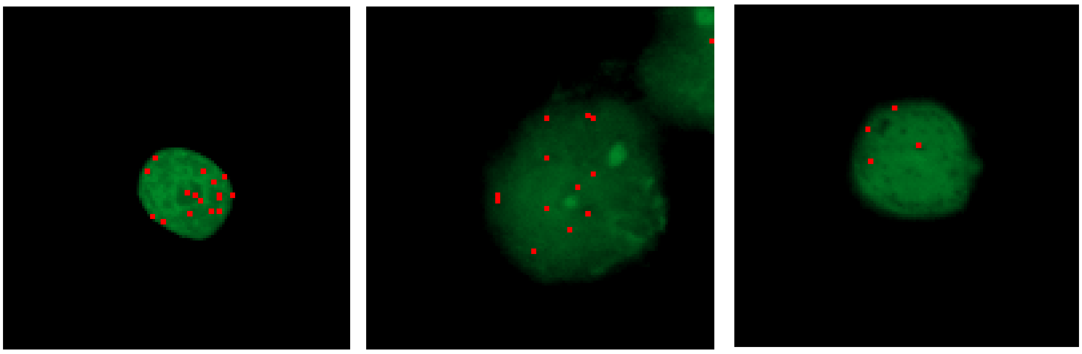

#47

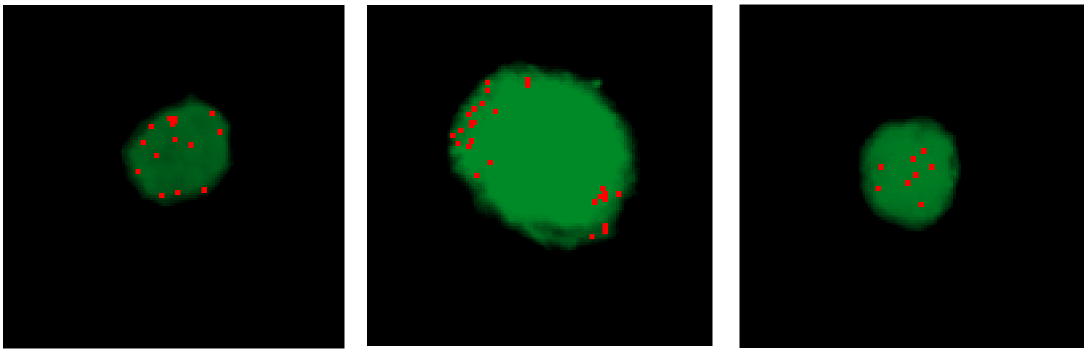

#48

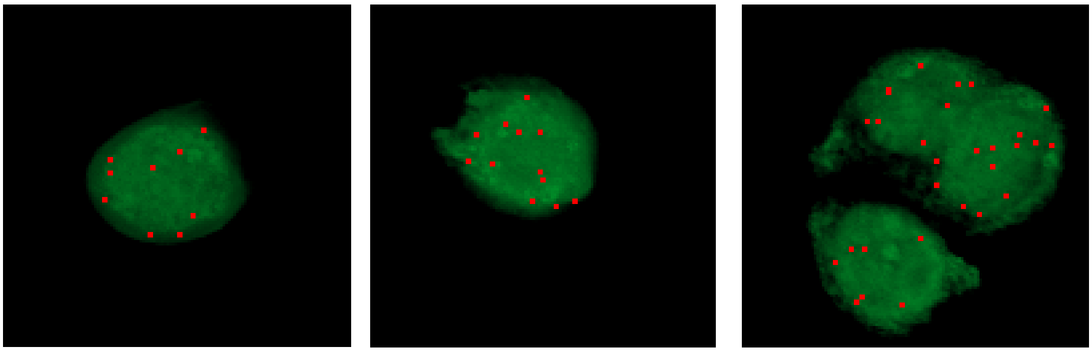

#59

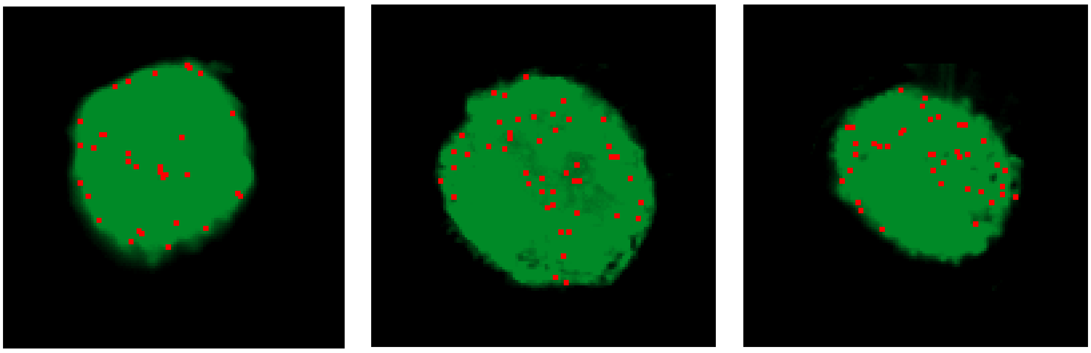

#62

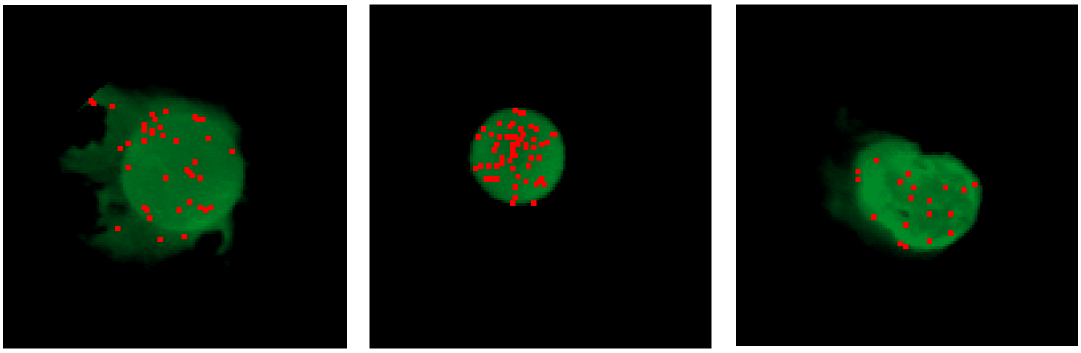

#65

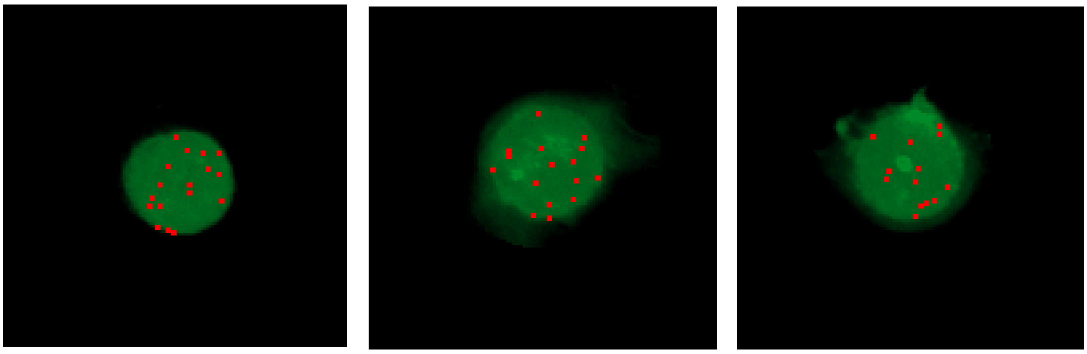

#68

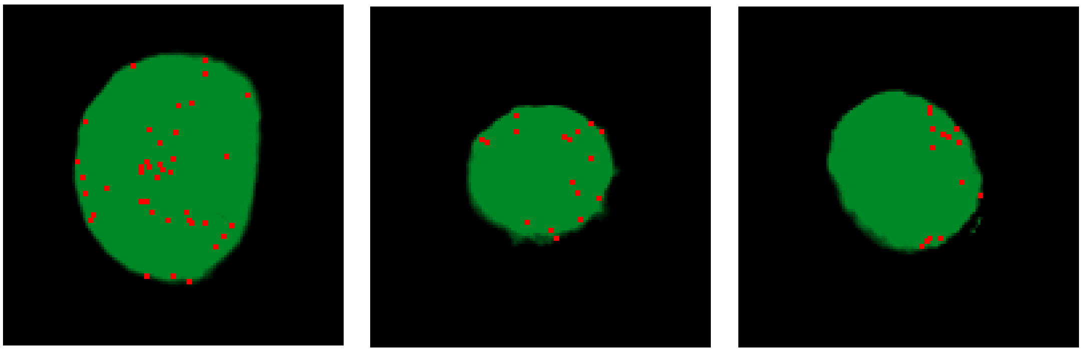

#70

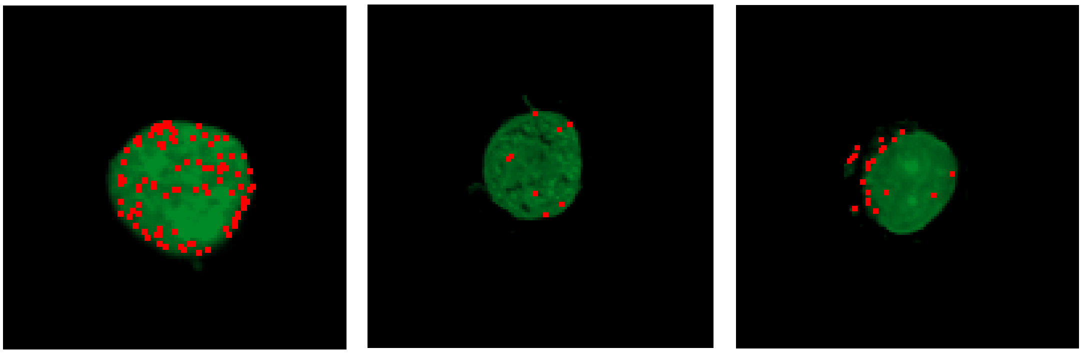

#71

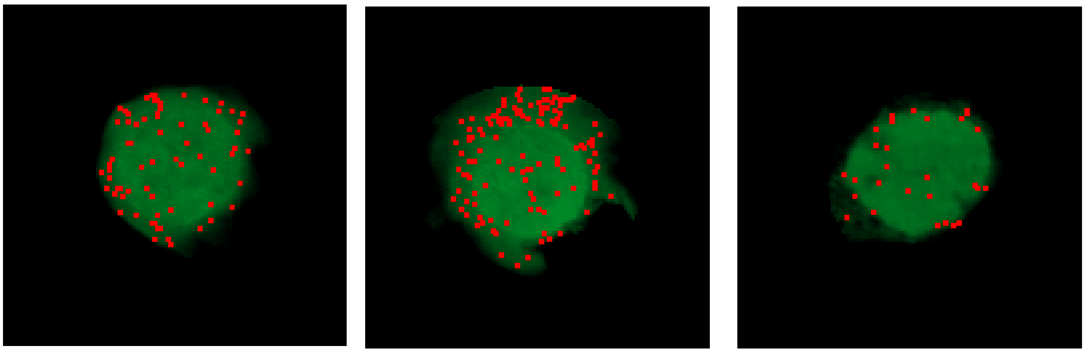

#72

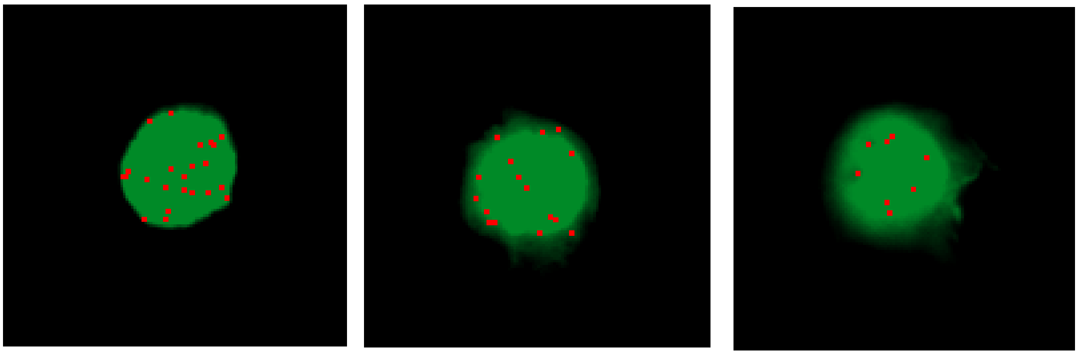

#75

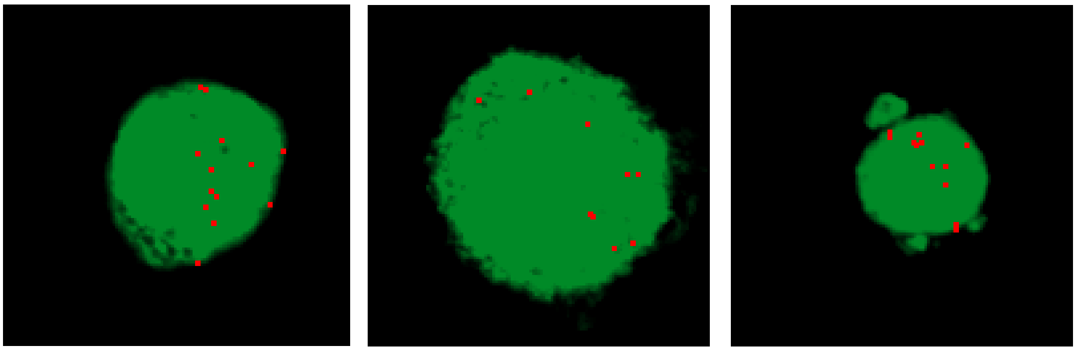

#89

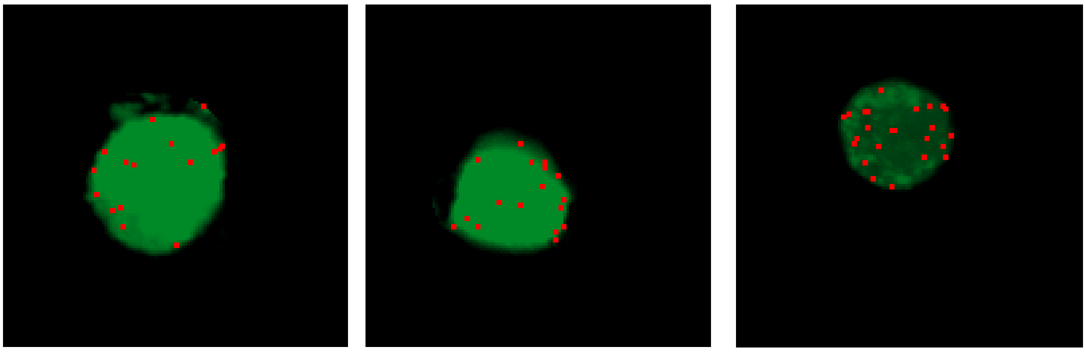

#94

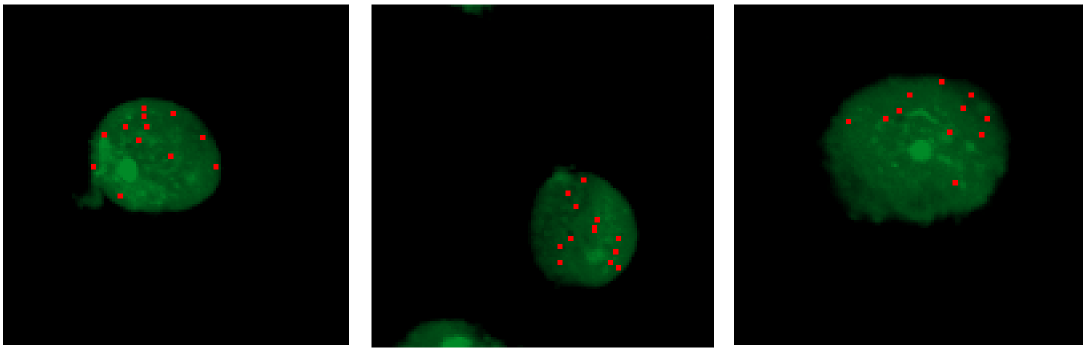

#101

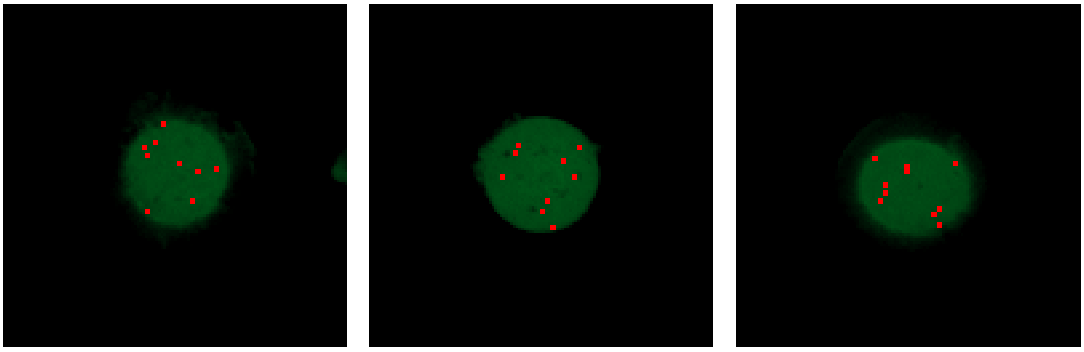

#134

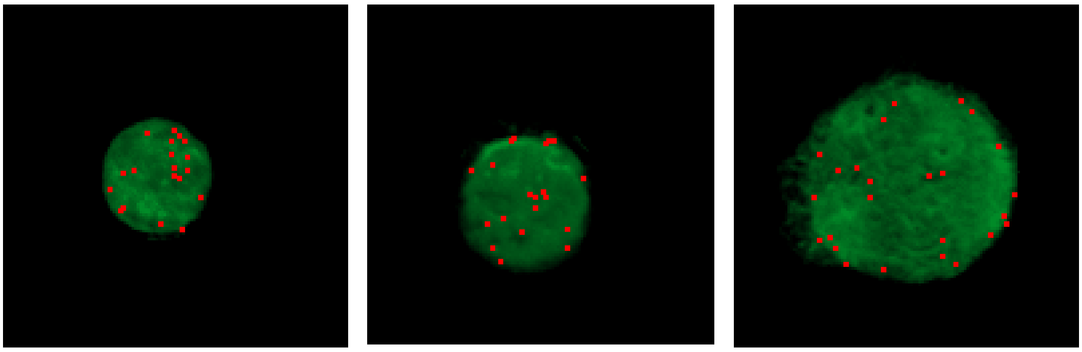

#145

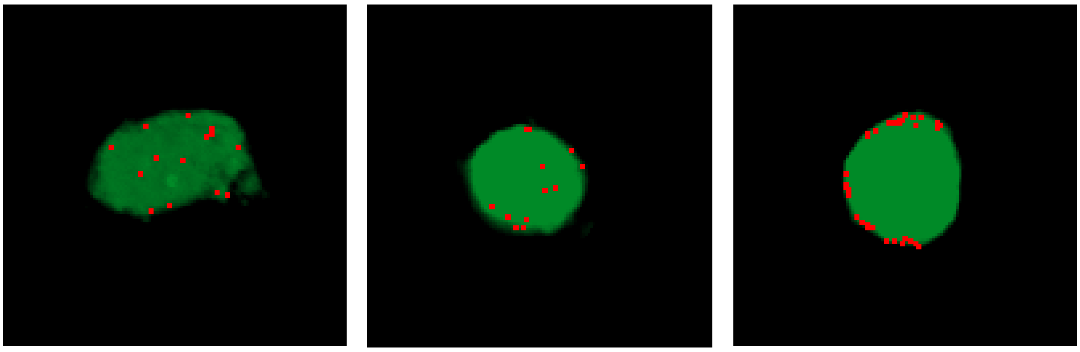

#232

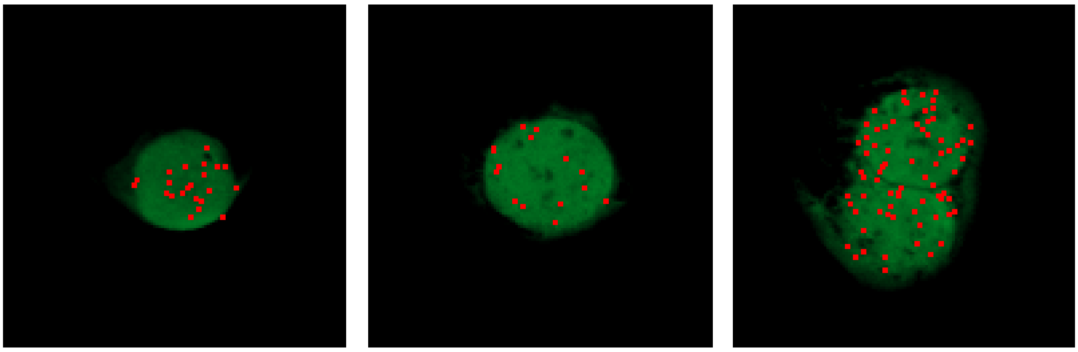

#257

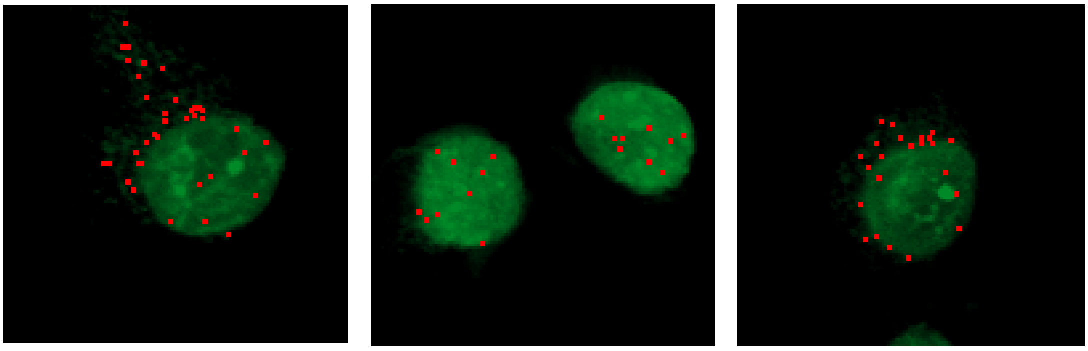

#259

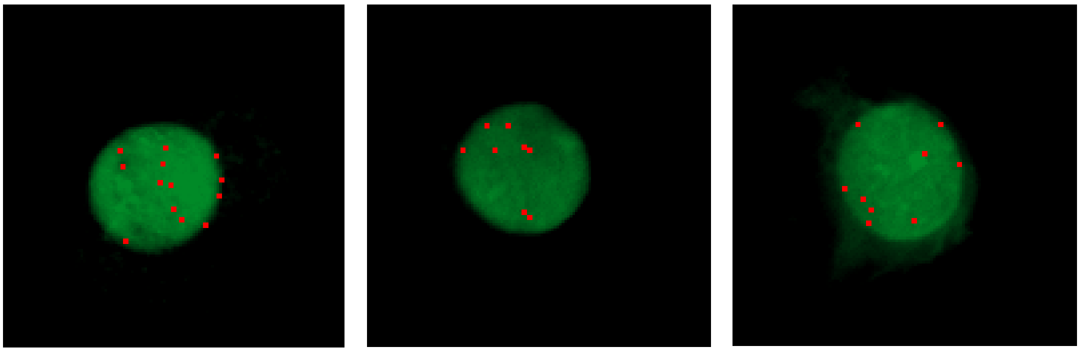

#261

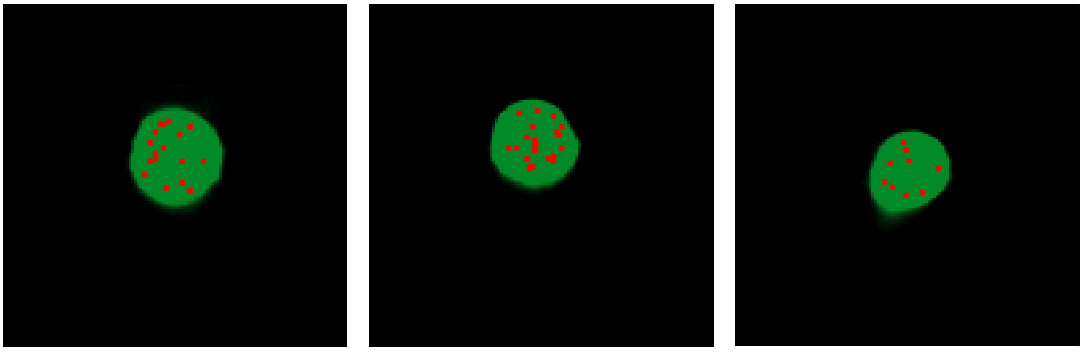

#263

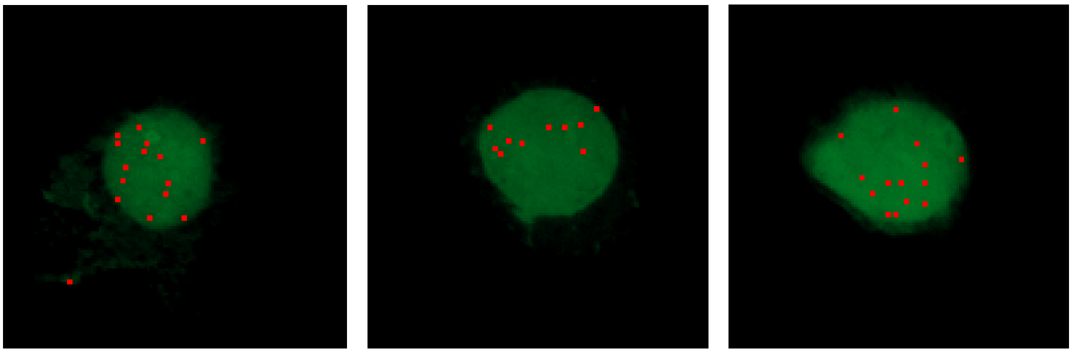

#265

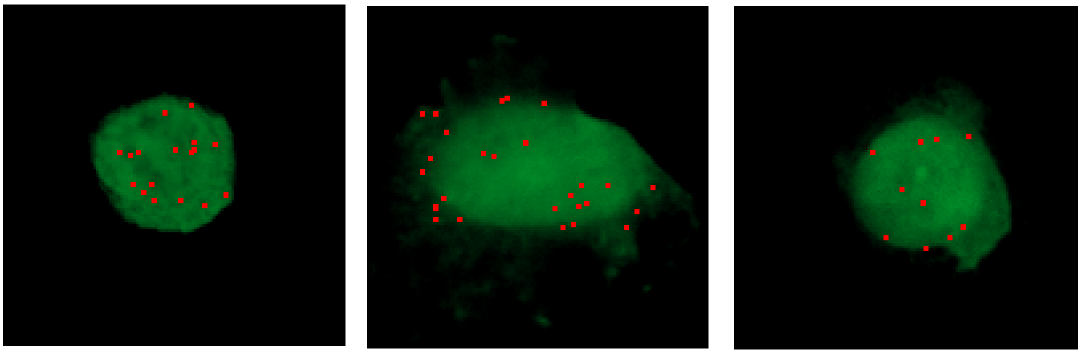

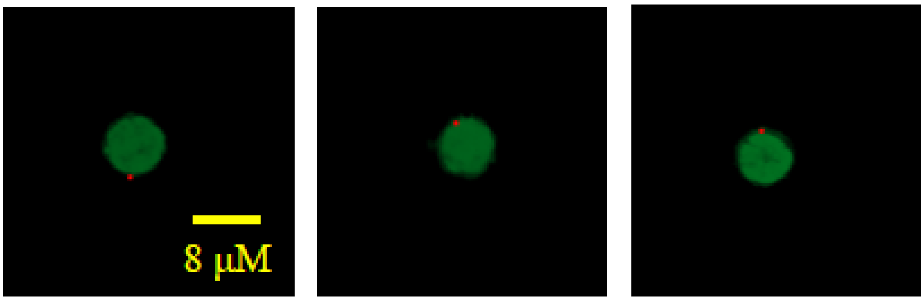

Figure S2. Binary FLIM images of the *o*-BMVC test for case #271: a typical nondiagnostic case because the spot size is relatively small.
